# Pangenome analysis of the genus *Herbiconiux* and proposal of four new species associated with Chinese medicinal plants

**DOI:** 10.3389/fmicb.2023.1119226

**Published:** 2023-02-28

**Authors:** Yang Deng, Zhu-Ming Jiang, Xue-Fei Han, Jing Su, Li-Yan Yu, Wei-Hong Liu, Yu-Qin Zhang

**Affiliations:** ^1^Institute of Medicinal Biotechnology, Chinese Academy of Medical Sciences and Peking Union Medical College, Beijing, China; ^2^State Key Laboratory of Dao-di Herb, Beijing, China; ^3^Yunnan Provincial Key Laboratory of Entomological Biopharmaceutical R&D, Dali University, Dali, China

**Keywords:** *Herbiconiux*, pangenome, polyphasic taxonomy, medicinal plants, plant growth-promotion

## Abstract

Five Gram-stain-positive, aerobic, non-motile actinobacterial strains designated as CPCC 205763^T^, CPCC 203386^T^, CPCC 205716^T^, CPCC 203406^T^, and CPCC 203407 were obtained from different ecosystems associated with four kinds of Chinese traditional medicinal plants. The 16S rRNA gene sequences of these five strains showed closely related to members of the genus *Herbiconiux* of the family *Microbacteriaceae*, with the highest similarities of 97.4–99.7% to the four validly named species of *Herbiconiux*. In the phylogenetic trees based on 16S rRNA gene sequences and the core genome, these isolates clustered into the clade of the genus *Herbiconiux* within the lineage of the family *Microbacteriaceae.* The overall genome relatedness indexes (values of ANI and dDDH) and the phenotypic properties (morphological, physiological and chemotaxonomic characteristics) of these isolates, readily supported to affiliate them to the genus *Herbiconiux*, representing four novel species, with the isolates CPCC 203406^T^ and CPCC 203407 being classified in the same species. For which the names *Herbiconiux aconitum* sp. nov. (type strain CPCC 205763^T^ = I19A-01430^T^ = CGMCC 1.60067^T^), *Herbiconiux daphne* sp. nov. (type strain CPCC 203386^T^ = I10A-01569^T^ = DSM 24546^T^ = KCTC 19839^T^), *Herbiconiux gentiana* sp. nov. (type strain CPCC 205716^T^ = I21A-01427^T^ = CGMCC 1.60064^T^), and *Herbiconiux oxytropis* sp. nov. (type strain CPCC 203406^T^ = I10A-02268^T^ = DSM 24549^T^ = KCTC 19840^T^) were proposed, respectively. In the genomes of these five strains, the putative encoding genes for amidase, endoglucanase, phosphatase, and superoxidative dismutase were retrieved, which were classified as biosynthetic genes/gene-clusters regarding plant growth-promotion (PGP) functions. The positive results from IAA-producing, cellulose-degrading and anti-oxidation experiments further approved their potential PGP bio-functions. Pangenome analysis of the genus *Herbiconiux* supported the polyphasic taxonomy results and confirmed their bio-function potential.

## Highlights

- The members of the genus *Herbiconiux* were mostly found to inhabit the niches associated with plants.
- However, the microbe-plant interaction still has not been investigated exhaustively due to lack of enough *Herbiconiux* strains as materials. In this study, five strains designated as CPCC 205763^T^, CPCC 203386^T^, CPCC 205716^T^, CPCC 203406^T^, and CPCC 203407 were obtained from different ecosystems associated with four kinds of Chinese traditional medicinal plants.
- The 16S rRNA gene sequences information and the values of ANI and dDDH by comparing their genome sequences together with the phenotypic (morphological, physiological and chemotaxonomic) properties, readily supported to affiliate them to the genus *Herbiconiux* representing four novel species.
- In the genomes of these five strains, we detected the encoding genes for aldehyde dehydrogenase (EC 1.2.1.3) and amidase (EC 3.5.1.4) coding gene *amiE* involved in indole-3-acetic acid (IAA) biosynthesis pathway, furthermore, experiments confirmed that these strains produced IAA, an important hormone for plant growth.
- As a reward, these five strains exhibited anti-oxidation ability, which we proposed to be endowed by these anti-inflammatory herbs that shared the common ecosystems with these strains. Our study primarily accumulated valuable actinobacterial resources to exemplify microbe-plant interaction.

## Introduction

Anti-inflammatory herbs have been widely used in Chinese typical ethnic drugs. Even nowadays, these ethnic drugs have still contributed significantly to the health of the people living in the marginal impoverished regions. We supposed that microorganisms associated with the anti-inflammatory herbs may gain some potential abilities to produce anti-inflammatory substances resulted from their co-evolution with these plants. During screening of the microorganisms with anti-inflammatory activities, five *Herbiconiux* spp. were collected. We carried out polyphasic taxonomic study on these strains. The detailed phenotypic and genotypic properties of these strains supported us to propose four novel species of the genus *Herbiconiux.*

Previously, *Herbiconiux* spp. were classified as members of the genus *Leifsonia* within the family *Microbacteriaceae* (Park et al., 1994). Later, combined the phylogenetic analysis based on 16S rRNA gene sequences and the chemotaxonomic characterization, Behrendt et al. (2011) reclassified *Leifsonia ginsengi* as *Herbiconiux ginsengi*. In 2012, two more species, *Herbiconiux moechotypicola* and *Herbiconiux flava* were identified, and accordingly, the description of the genus was emended (Kim et al., 2012). Currently, four species with valid scientific names have been accommodated in the genus *Herbiconiux*,^1^ sharing the taxonomic characteristics as follows. The peptidoglycan is of the B2γ type with D-and L-2,4-diaminobutyric acid as the diagnostic diamino acids, and glycine, alanine and *threo*-3-hydroxyglutamic acid may present. The predominant menaquinone is MK-11; major amounts of MK-10 may also be present. Cellular fatty acids mainly comprise *iso-and anteiso*-branched fatty acids, with *anteiso*-C_15:0_ as a major component. Cyclohexyl-C_17:0_ may be present. The DNA G + C content is 69–71% (Hamada et al., 2012).

It is so interesting that except the type strain of *H. moechotypicola,* all of the type strains of other three species of the genus *Herbiconiux* were isolated from the ecosystems associated with plants. In this study, five more isolates representing four novel *Herbiconiux* species were obtained from the ecosystems involved in anti-inflammatory herbs.

The primary goal of the present research was to study the properties of *Herbiconiux* members inhabiting Chinese traditional medicinal plants relating niches, to explore the genotypic properties regarding plant growth-promotion functions. As a result, in the genomes of strains CPCC 205763^T^ representing *Herbiconiux aconitum* sp. nov., CPCC 203386^T^ representing *Herbiconiux daphne* sp. nov., CPCC 205716^T^ representing *Herbiconiux gentiana* sp. nov., and CPCC 203406^T^ and CPCC 203407 representing *Herbiconiux oxytropis* sp. nov., putative encoding genes for endoglucanase, phosphatase, superoxidative dismutase and amidase were retrieved, and the corresponding phenotypic experiments further validated the putative functions. On the one hand, the microbes harbor the abilities to synthesize plant growth hormone, indole-3-acetic acid (IAA), remove excessive harmful oxygen negative ions, transform the substrates that are hard to be assimilated directly by plants, to promote plant growth; on the other hand, these anti-inflammatory plants may horizontally transfer the abilities to produce anti-inflammatory substances to the microorganisms. Our study preliminarily exemplified microbe-plant interaction.

## Materials and methods

### Collection of samples, isolation and identification of *Herbiconiux* strains

The rhizosphere soil sample attached to the medicinal plant *Aconitum carmichaelii* was collected from Yili Valley (42^°^34′37′′ N, 81^°^13′19′′ E, 1,878 mH), Xinjiang Province, north-west China, labeled as IMB15132S; the rhizosphere soil sample of the plant *Gentiana rigescens* was collected from Leigong Mountain (26^°^23′38′′ N, 107^°^35′49′′ E, 2,071 mH), Kaili, Guizhou Province, south-west China (labeled as IMB20178S). The herb of *Daphne aurantiaca* labeled as IMB12384P was obtained from the Shangrila Alpine Botanical Garden (28^°^39′18′′ N, 100^°^18′19′′ E, 3,343 mH), Yunnan Province, south-west China. The leaf of a medicinal plant *Oxytropis falcata* (labeled as IMB11009P) was collected from Bujiangda county (29^°^55′47′′ N, 93^°^12′41′′ E, 3,992 mH), Tibet, west China.

The rhizosphere soil samples IMB15132S and IMB20178S were put into the sterilized envelopes and then taken to the laboratory within 1 week after collection. Isolation of microorganisms from these soils was carried out following the procedure described by Jiang et al. (2021). The medicinal plant samples IMB12384P and IMB11009P were sealed with sterile wax at the incisions before they were sent to the laboratory. The follow-up work was performed as described by Deng et al. (2022) to acquire the endophytic bacterial strains. Distinct colonies picked from the isolation plates were streaked into newly prepared PYG agar plates (g l^−1^; peptone 3, yeast extract 5, glycerol 10, betaine hydrochloride 1.25, sodium pyruvate 1.25, agar 15, pH 7.2), respectively. The purified isolates were maintained on PYG slants at 4°C and also as glycerol suspensions (20%, v/v) at –80°C.

The *Herbiconiux* spp. were primarily identified according to the 16S rRNA gene sequence comparison following next steps. Genomic DNA was extracted according to the manual of a commercial genomic DNA extraction kit (TianGen, China). PCR amplification of the strains’ 16S rRNA genes was carried out using the bacterial universal primers 27F and 1492R (Li et al., 2007). The obtained sequences were compared with available 16S rRNA gene sequences from GenBank using the BLAST program and the EzTaxon-e server^2^ to determine an approximate taxonomic affiliation (Yoon et al., 2017a). Multiple sequence alignment and analysis of the data were performed by using the molecular evolutionary genetics analysis (MEGA) software package version X (Kumar et al., 2018). The phylogenetic trees were reconstructed by the software package MEGA version X using the neighbor-joining (Kimura, 1979), and confirmed by maximum-likelihood (Felsenstein, 1981) and maximum-parsimony (Kluge and Farris, 1969) tree-making methods. Bootstrap analysis with 1,000 replicates was performed to obtain the confidence level of the branches (Felsenstein, 1985).

The reference strains of *Herbiconiux ginsengi* KCTC 19440^T^ and *Herbiconiux moechotypicola* KCTC 19653^T^ were obtained from KCTC (Korean Collection for Type Cultures), and *Herbiconiux flava* NBRC 16403^T^ and *Herbiconiux solani* NBRC 106740^T^ were acquired from NBRC (NITE Biological Resource Center), respectively, and they were included in some assays in parallel.

### Polyphasic taxonomic study

#### Morphological and physiological characterization

Growth conditions of the strains were tested using ISP 2 (Shirling and Gottlieb, 1966), Tryptic soy agar (TSA, Difco), Reasoner’s 2A agar (R2A, Difco), nutrition agar (NA) and PYG agar. The growth of the strains was monitored at 4, 10, 15, 20, 28, 30, 32, 35, 37 and 40°C using TSB medium for cultivation for 2 weeks. The pH range (5.0–11.0, at intervals of 1 pH unit) for growth was observed in TSB using the buffer system described by Xu et al. (2005). Tolerance to NaCl was examined using TSB as the basal medium with different total NaCl concentrations [0–10% (w/v; at 1% intervals)]. Colony characteristics were recorded after 3-day incubation on TSA at 28°C. The Gram reaction was tested by the Gram-staining method as described by Magee et al. (1975), and the capsule-staining was performed according to the protocols described previously (Breakwell et al., 2009). The cells were observed using light microscopy (BH-2, Olympus). Motility of cells was examined on TSA semi-solid agar medium (0.3%, w/v) and then double checked using hanging drop method (Bernardet et al., 2002). The morphological characteristics of the exponentially-growing cells were observed using transmission electron microscopy (JEOL JEM-1010).

The assimilation of carbon compounds by the isolates was tested at 28°C using Biolog GEN III Microplates and observed in an Omnilog device (BIOLOG Inc., Hayward, CA, United States). Other metabolic properties were examined by API 50CH, API 20NE and API ZYM test kits (bioMerieux) according to the manufacturer’s instructions. Results were evaluated after incubation at 28°C for 72–144 h. Activities of catalase, oxidase and urease, hydrolysis of Tweens and starch were investigated according to the procedures as previously described (Zhou et al., 1998; Yuan et al., 2008). The cellulose degradation activity was examined using CMC-Na screening medium (Reinhold-Hurek et al., 1993). The ability of the strains to produce IAA (indole-3-acetic acid) was tested using colorimetric methods (Bric et al., 1991) and recorded as described by Jiang et al. (2022). The scavenging effect of these strains on DPPH (2,2-diphenyl-1-picrylhydrazyl) radical was studied following the method described earlier (Singh and Rajini, 2004) and slightly modified according to the manufacture’s instruction of DPPH scavenging kit (Yuanye Bio-Technology Co., Ltd., Shanghai, China) as follows. Cells were harvested and washed with 0.1 M PBS solution for twice, and then suspended in 0.1 M PBS solution to the final concentration of 10^5^ cells ml^−1^. The cells were sonicated in ice bath at 200 W for 3 s, followed by 10 s break. Repeated 30 rounds. The broken cells were discarded by centrifugation at 10000 r.p.m. for 10 min to collect the supernatant. Then added 50 μl of the supernatant into 450 μl of DPPH solution (0.1 mM in 95% ethanol) and incubated the mixture in dark at room temperature for 30 min. The absorbance of the resulting solution was read at 517 nm against a blank. The scavenging activity of vitamin C standard solution (2, 4, 6, 8, 10 mg L^−1^) was taken as the abscissa and the absorbance value of the corresponding concentration at 517 nm as the ordinate to draw a scatter diagram, and a linear trend line was added to obtain the scavenging activity of vitamin C standard curve. The radical scavenging activity was measured as a decrease in the absorbance of DPPH and was calculated using the following equation:

$$\mathrm{Scavenging activity}\;\left(\%\right)=\left[1-\left(A_{s}-A_{b}\right)/A_{c}\right]\times 100$$

A_b_, A_c_ and A_s_ is the absorbance of the blank (PBS), control (ethanol) and the sample, respectively.

#### Chemotaxonomic properties

Biomass for chemotaxonomic studies of the strains was obtained by cultivation in flasks on a rotary shaker (180 r.p.m.) using GYM broth at 28°C for 4 days except that cellular fatty acids extraction and analysis were conducted using the cultures harvested from Tryptic soy broth (TSB). The diagnostic isomers of diaminopimelic acid in the whole cell hydrolysates (4 N HCl, 100°C, 15 h) of these strains were subjected to thin-layer chromatography on cellulose plates using the solvent system of Schleifer and Kandler (1972). The sugar analysis of the whole cell hydrolysates followed procedures described by Staneck and Roberts (1974). Polar lipids were extracted and examined by two-dimensional TLC and identified using previously described procedures (Minnikin et al., 1984). Menaquinones were isolated using the method of Collins et al. (1977) and were analyzed by HPLC (Groth et al., 1997). Analysis of the cellular fatty acid pattern followed the described methods using the MIDI system (Microbial ID, Inc., Newark, Del; Kroppenstedt, 1985; Meier et al., 1993). MIDI Sherlock Version 6.0 and ACTIN1 database were employed for the analysis.

### Genomic traits

#### Genome sequencing and assembly

The whole-genome sequencing was implemented using an Illumina HiSeq 4,000 system (Illumina, SanDiego, CA, United States) at the Beijing Genomics Institute (Beijing, China). Genomic DNA was sheared randomly to construct three read libraries with length of 300 bp by a Bioruptor ultrasonicator (Diagenode, Denville, NJ, United States) and physico-chemical methods. The paired-end fragment libraries were sequenced according to the Illumina HiSeq 4,000 system’s protocol. Raw reads of low quality from paired-end sequencing (those with consecutive bases covered by fewer than five reads) were discarded. The sequenced reads were assembled using SOAPdenovo v1.05 software. Digital DNA–DNA hybridization (dDDH) and average nucleotide identity (ANI) values between these strains and other related strains were calculated using the Genome-to-Genome Distance Calculator (GGDC, version 3.0; http://ggdc.dsmz.de/ggdc.php; Auch et al., 2010) and with the ezbiocloud platform (Yoon et al., 2017b), respectively.

#### Genome component prediction

The assembled genomic sequences of strains CPCC 205763^T^, CPCC 203386^T^, CPCC 205716^T^, CPCC 203406^T^, CPCC 203407 and *H. moechotypicola* KCTC 19653^T^ were predicted by glimmer3^3^ with Hidden Markov models and were functional annotated by the KEGG database (Kyoto Encyclopedia of Genes and Genomes). The assembled genomic sequences of other eight *Herbiconiux* strains (*H. flava* DSM 26474^T^, *H. ginseng* CGMCC 4.3491^T^, *H. solani* NBRC 106740^T^, *Herbiconiux* sp. L3-i23, *Herbiconiux* sp. SALV-R1, *Herbiconiux* sp. SYSU D00978, *Herbiconiux* sp. VKM Ac-1786 and *Herbiconiux* sp. VKM Ac-2,851) were downloaded from NCBI database and were also corrected as functionally annotated by the KEGG database. The Interpro database^4^ and the UniProt database^5^ were used for validation of putative functional genes. The tRNAscan-SE (Lowe and Eddy, 1997), RNAmmer, and the Rfam databases were employed for sorting of tRNA, rRNA and sRNAs, respectively.

#### Analysis of Pan-genome, functional genes and biosynthetic gene clusters

The protein sequences from each genome were followed by functional genes retrieval and pan-genome analysis. For pathway analysis, the predicted proteins sequences were uploaded to KEGG Automatic Annotation Server. Bacterial Pan-genome Analysis (BPGA) pipeline was applied for analysis of the genomic diversity of the *Herbiconiux* population. Pan-genome analysis was performed by BPGA 1.3 using default settings (Chaudhari et al., 2016). Predictions of gene clusters for natural products were performed using antiSMASH (antibiotic & Secondary Metabolite Analysis Shell, http://antismash.secondarymetabolites.org; Blin et al., 2021).

## Results and analysis

### Phenotypic characteristics

Strains designated as CPCC 205763^T^ and CPCC 205716^T^ were recovered from herb rhizospheric-niche samples IMB15132S and IMB20178S, respectively. Plant endophytic strains CPCC 203386^T^, CPCC 203406^T^, and CPCC 203407 were isolated from the samples IMB12384P (the stem of a medicinal plant *Daphne aurantiaca*) and IMB11009P (the leaves of *Oxytropis falcata*), respectively.

Good growth of these five isolates was observed on tested media ISP 2, TSA, R2A, and PYG, at 28–32°C and pH 7.0. NaCl was not required for growth. Light to bright yellow colonies formed on TSA with diameter of 0.8–1.2 mm in diameter. Capsulated cells were Gram-staining-positive, non-motile and rod-shaped. Detailed physiological and biochemical characteristics results from Biolog and API assay kits were listed in Table 1 and species description.

**Table 1:** Differential characteristics between strains CPCC 205763^T^, CPCC 203386^T^, CPCC 205716^T^, CPCC 203406^T^, CPCC 203407 and the closely related type strains of the genus *Herbiconiux*.

Characteristic	1	2	3	4	5	6	7	8	9
Isolation source	rhizosphere soil of the plant *Aconitum carmichaelii*	root of *Ginseng* ^a^	stem of a medicinal plant *Daphne aurantiaca*	phyllosphere of *Sedge* ^b^	rhizosphere soil of the plant *gentiana dipteris*	leaf of a medicinal plant *Oxytropis falcata*	leaf of a medicinal plant *Oxytropis falcata*	phyllosphere of potato ^c^	guts of *Moechotypa diphysis* ^d^
Temperature range (°C)	10–37	10–37	10–37	4–37	4–42	10–37	10–37	10–28	10–37
pH range	7–8	6–8	7–8	7–8	7–8	7–8	7–8	4–8	7–8
NaCl Tolerance (%, w/v)	0–2	0–2	0–3	0–3	0–3	0–3	0–2	0–3	0–4
Hydrolysis of									
gelatin	−	−	−	+	−	+	+	−	+
Produce acids from (API 50CH)									
L-Arabinose	+	+	+	+	−	+	+	+	−
D-Ribose	+	+	+	−	−	+	+	+	−
D-Xylose	+	+	+	+	W	+	+	+	−
L-Xylose	−	−	W	−	W	W	−	−	−
D-Fructose	+	+	W	+	+	+	+	+	+
Sorbitol	−	−	−	−	−	−	−	−	+
Laetrile	−	+	−	+	+	−	−	+	−
Salicin	W	+	+	+	−	−	−	+	−
D-Cellobiose	+	+	+	+	+	+	+	+	−
D-Galactose	+	−	W	−	−	−	−	−	−
D-Melibiose	−	−	−	−	+	−	−	−	−
D-Sucrose	+	+	+	+	+	+	+	+	−
D-Trehalose	+	+	+	+	+	+	+	-	−
Inulin	+	−	−	−	−	−	−	+	−
D-Melezitose	+	−	−	−	−	−	−	−	−
D-Raffinose	+	−	−	−	−	−	−	+	−
D-Gentianediose	−	+	−	+	+	−	−	+	−
D-Lyxose	+	+	−	+	+	+	W	−	−
D-Tagatose	+	+	−	+	+	−	−	−	−
D-Fucose	−	−	−	−	+	−	−	+	−
L-Fucose	−	−	+	+	+	−	−	+	−
Assimilation of (Biolog GEN III)									
Stachyose	+	−	−	+	−	−	−	+	−
Melisaccharide	+	−	−	+	−	−	−	+	−
*α*-D-Galactose	+	+	+	+	+	+	+	−	+
Melibiose	+	+	−	+	−	−	−	−	+
*β-*Formyl-D-glucoside	−	+	−	+	−	−	−	−	−
N-Acetyl-D-glucosamine	−	−	−	−	+	−	−	−	−
N-Acetyl-β-D-mannose amine	−	−	−	−	+	−	−	−	−
*α-D*-Glucose	+	+	+	−	+	+	+	+	+
D-Galactose	+	+	+	−	+	+	+	+	+
inosine	+	+	+	+	+	+	+	−	+
D-Sorbitol	+	−	−	−	−	−	−	−	−
D-Mannitol	+	+	+	−	+	+	+	+	+
D-Arabyl alcohol	+	+	−	+	+	+	+	−	−
Glycerin	+	+	+	−	+	+	+	+	+
D-Glucose-6-phosphate	−	−	−	−	−	−	−	+	−
D-Fructose-6-phosphate	−	−	−	+	−	−	−	+	−
L-Alanine	−	−	−	+	+	+	+	−	+
L-Glutamic acid	+	+	−	−	+	−	+	+	+
L-Pyroglutamic acid	−	−	−	+	−	−	−	−	−
D-Galacturonic acid	−	−	−	+	−	−	−	+	−
D-Gluconic acid	−	+	+	+	+	+	+	+	+
P-Hydroxyphenylacetic acid	−	+	+	−	−	−	−	−	−
D-Methyl lactate	−	−	−	+	−	−	−	−	−
L-Lactic acid	+	+	+	−	+	+	+	−	+
citric acid	−	−	−	−	+	+	+	−	−
*α*-Ketoglutaric acid	−	−	−	+	−	+	−	−	−
D-Malic acid	−	+	−	−	+	+	+	−	+
L-Malic acid	−	+	−	+	+	+	+	−	+
Twain 40	+	+	+	+	+	+	+	−	+
*α-*Hydroxybutyric acid	+	+	+	+	+	+	+	−	−
propionic acid	+	+	+	+	+	+	+	−	−
acetic acid	+	+	+	+	+	+	+	−	+
API ZYM									
Alkaline phosphatase	+	−	−	+	+	W	−	+	−
Esterase C4	+	−	+	+	+	+	+	W	W
Lipoid esterase C8	+	+	+	+	+	+	+	W	+
Valine arylaminase	+	+	+	+	+	+	+	−	W
Cystine arylaminase	+	−	W	+	+	+	+	−	−
Chymotrypsin	−	−	−	−	−	−	−	−	W
Acid phosphatase	+	W	+	+	+	+	+	+	+
Naphthol-AS-BI-phosphate hydrolase	+	+	+	+	+	+	+	W	+
*α*-Galactosidase	+	W	−	+	+	−	−	−	W
*β*-Galactosidase	+	+	+	+	+	−	−	−	−
*α*-Glucosidase	+	+	+	+	+	+	+	+	+
*β*-Glucosidase	+	+	+	+	+	+	+	+	+
N-Acetyl-glucosaminidase	−	−	−	−	−	−	−	−	−
*α*-Mannosidase	−	−	−	−	−	−	−	−	−
*β*-Fucosidase	−	−	−	−	−	−	−	−	−

Strains: 1, CPCC 205763^T^; 2, *H. ginsengi* KCTC 19440^T^; 3, CPCC 203386^T^; 4, *H. flava* NBRC 16403^T^; 5, CPCC 205716^T^; 6, CPCC 203406^T^; 7, CPCC 203407; 8, *H. solani* NBRC 106740^T^; 9, *H. moechotypicola* KCTC 19653^T^. Data were from this study except (a) Behrendt et al., 2011; (b) Hamada et al., 2012; (c) Behrendt et al., 2011; (d) Kim et al., 2012. +, positive; −, negative; W, weakly positive.

The clear transparent circle was observed around the strains on plate with CMC-Na as the sole carbon source, which demonstrated these strains were cellulose-degrading microbes. IAA was detected in the fermentation broth of strains CPCC 205763^T^, CPCC 203386^T^, CPCC 205716^T^, CPCC 203406^T^, and CPCC 203407. As shown in Supplementary Figure S1, the linear regression equation y = 0.0255x + 0.0411, r^2^ = 0.9992, gave a good fit. Accordingly, the IAA content produced by the test strains was calculated. The IAA content yielded from the fermentation broth of CPCC 205763^T^, CPCC 203386^T^, CPCC 205716^T^, CPCC 203406^T^, and CPCC 203407 was 5.89 ± 0.91 mg L^−1^, 0.94 ± 0.00 mg L^−1^, 4.61 ± 0.02 mg L^−1^, 2.17 ± 0.02 mg L^−1^ and 2.10 ± 0.05 mg L^−1^, respectively. Total DPPH scavenging potential of the test strains was measured and depicted in Supplementary Figure S2. The linear regression equation of the radical scavenging activity of vitamin C standard curve y = 11.65x + 17.724, r^2^ = 0.9999, had a good fit. Strains CPCC 205763^T^, CPCC 203386^T^, CPCC 205716^T^, CPCC 203406^T^ and CPCC 203407 were capable of neutralizing the DPPH free radicals *via* hydrogen donating activity by 38.3 ± 0.9%, 78.6 ± 5.8%, 51.8 ± 3.3%, 65.4 ± 10.9%, and 33.8 ± 4.1% at the concentration of 10^5^ cells mL^−1^, respectively. Accordingly, the *Herbiconiux* strains harbored a higher antioxidant activity than that of 4 mg L^−1^ vitamin C.

### Chemotaxonomy

In the cell-wall peptidoglycan of these five strains, 2,4-diaminobutyric acid was detected as the diagnostic diamino acid. Diphosphatidylglycerol (DPG), phosphatidylglycerol (PG) and glycolipid (GL) were present in the polar lipids extract of the five strains (Supplementary Figure S3). The predominant respiratory quinone was detected as MK-11. In the acid profiles, *anteiso*-C_15:0_ was the major component (26–70%). In addition to *anteiso*-C_15:0_, strain CPCC 205763^T^ contained C_18_: _1_*ɷ7c*/C_18: 1_*ɷ6c* (28.1%), strain CPCC 203386^T^ contained C_17:1_ *ɷ*9c (25.0%) and *anteiso*-C_17:0_ (11.1%), CPCC 205716^T^ contained *iso*-C_16:0_ (20.6%), CPCC 203406^T^ and CPCC 203407 contained *anteiso*-C_17:0 and_ *iso*-C_16:0_ as the major fatty acids, respectively (Supplementary Table S1).

### Phylogenetic analysis

**Figure 1:**
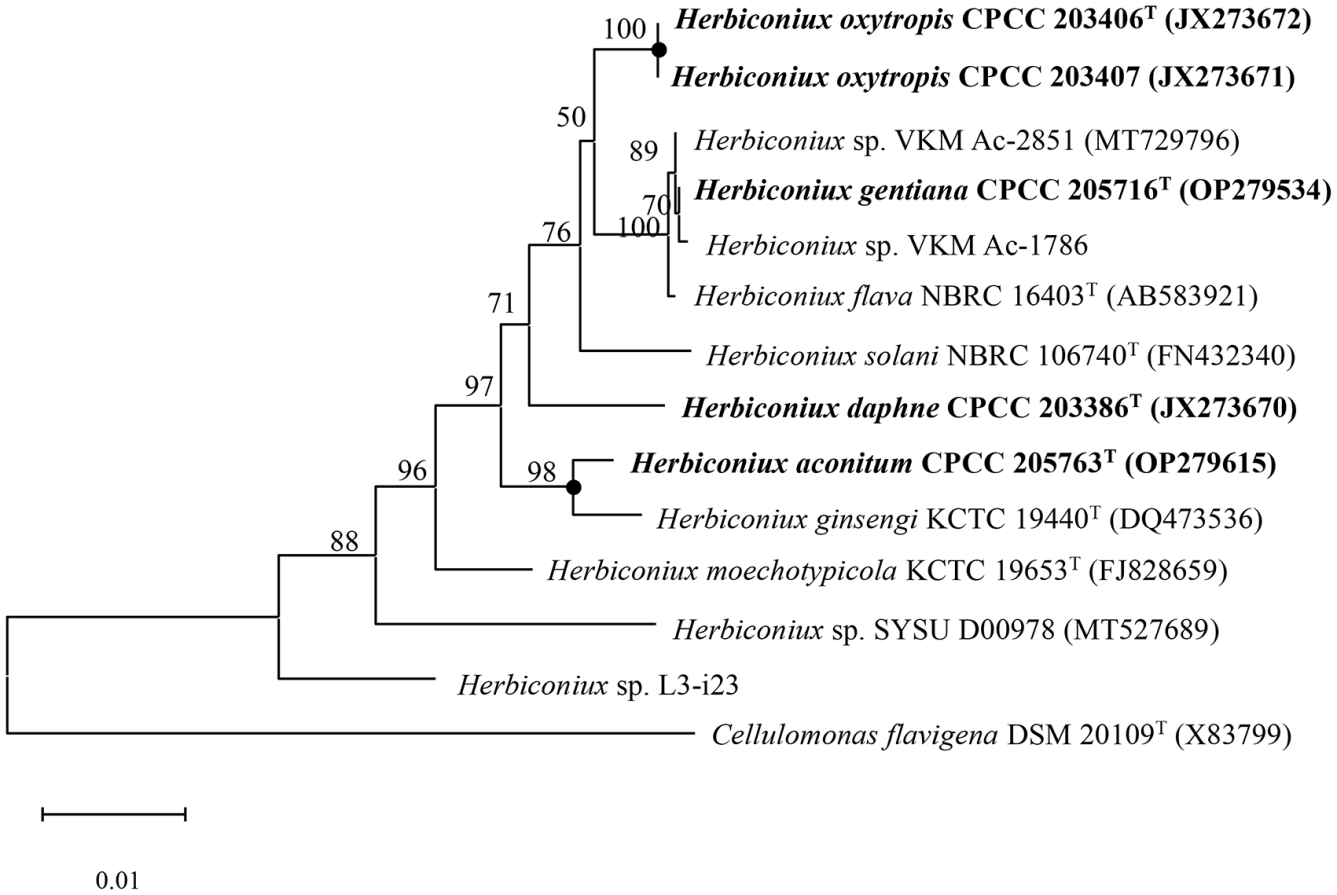
Neighbour-joining phylogenetic tree based on 16S rRNA gene sequences of strains CPCC 205763^T^, CPCC 203386^T^, CPCC 205716^T^, CPCC 203406^T^, CPCC 203407, and related species of the genus *Herbiconiux*. Filled circles indicate that the corresponding nodes were also recovered in the trees generated with the maximum-likelihood and maximum-parsimony methods. Bootstrap values (those above 50%) are shown as percentages of 1,000 replicates. *Cellulomonas flavigena* DSM 20109^T^ (GenBank accession no. X83799) was used as an outgroup. Bar, 0.01 nt substitution per nt.

**Figure 2:**
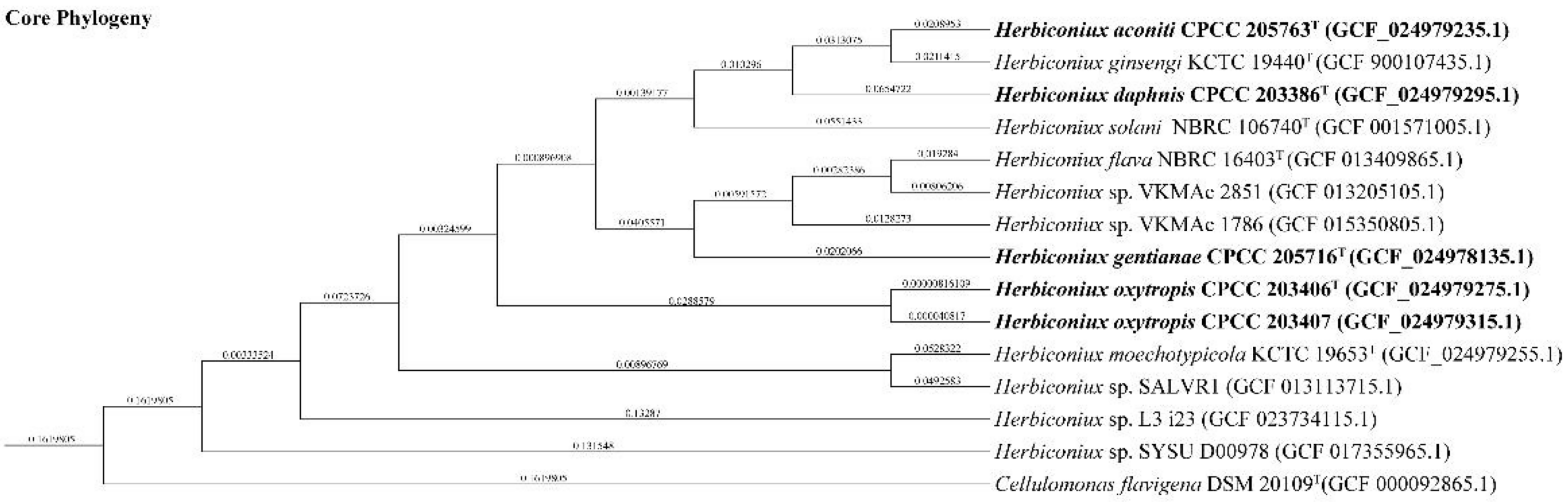
Phylogenetic tree constructed by BPGA showing the relationship of the newly proposed species with other species of the genus *Herbiconiux* based on concatenated core genes.

The almost full-length 16S rRNA gene sequences (1,512 bp, 1,521 bp, 1,515 bp, 1,511 bp and 1,511 bp) of strains CPCC 205763^T^, CPCC 203386^T^, CPCC 205716^T^, CPCC 203406^T^, CPCC 203407 were obtained. The alignment results of the 16S rRNA gene sequences indicated that the five strains were member of the family *Microbacteriaceae* with closely related to the genus *Herbiconiux*. The 16S rRNA gene sequence of the strain CPCC 205763^T^ showed the highest similarity with *H. ginseng* KCTC 19440^T^ (99.2%), *H. solani* NBRC 106740^T^ (98.3%), *H. moechotypicola* RB-62^T^ (98.2%), *H. flava* NBRC 16403^T^ (97.9%) and no more than 97.5% similarities with other type strains of the family *Microbacteriaceae*. The 16S rRNA gene sequences of the strains CPCC 203386^T^, CPCC 205716^T^, CPCC 203406^T^, CPCC 203407 showed highest similarities with *H. flava* NBRC 16403^T^ (98.3–99.9%), *H. ginseng* KCTC 19440^T^ (97.7–98.3%), *H. moechotypicola* RB-62^T^ (97.7–98.1%) and *H. solani* NBRC 106740^T^ (97.8–98.8%; Supplementary Table S2). In the phylogenetic tree based on the 16S rRNA gene sequences, these five strains fell in the genus *Herbiconiux* lineage. The strain CPCC 205763^T^ formed a robust unique cluster with *H. ginsengi* KCTC 19440^T^, and the strains CPCC 203386^T^, CPCC 205716^T^, CPCC 203406^T^ and CPCC 203407 formed a stable branch with the strains *H. flava* NBRC 16403^T^, *H. solani* NBRC 106740^T^ and two unclassified strains of the genus *Herbiconiux* in the neighbor-joining tree (Figure 1), which showed almost the same case in the maximum-parsimony tree, maximum-likelihood tree and phylogenetic trees based on the concatenated core genes (Figure 2) and pan-matrix by pan-genome analysis (Supplementary Figure S4).

The genomic G + C content of these five strains ranged in 68.6–71.0%. ANI values calculated between strains CPCC 205763^T^, CPCC 203386^T^, CPCC 205716^T^, CPCC 203406^T^, and other type strains of *Herbiconiux* species were all less than 89.2%, and the corresponding dDDH values were below 68.1% (Supplementary Table S3). These values were lower than the thresholds used to delineate bacterial species (i.e., ANI < 95% and dDDH <70%; Kim et al., 2012). While strains CPCC 203407 and CPCC 203406^T^ shared a high ANI value of 100% and a high dDDH value of 99.9% (Supplementary Table S3), which were consistent with the high level of 16S rRNA gene sequence similarity (100%) between the two strains, consistently indicating the assignment of these two strains to a same species of the genus *Herbiconiux* (Kim et al., 2012).

Filled circles indicate that the corresponding nodes were also recovered in the trees generated with the maximum-likelihood and maximum-parsimony methods. Bootstrap values (those above 50%) are shown as percentages of 1,000 replicates. *Cellulomonas flavigena* DSM 20109^T^ (GenBank accession no. X83799) was used as an outgroup. Bar, 0.01 nt substitution per nt.

### Genome features

**Figure 3:**
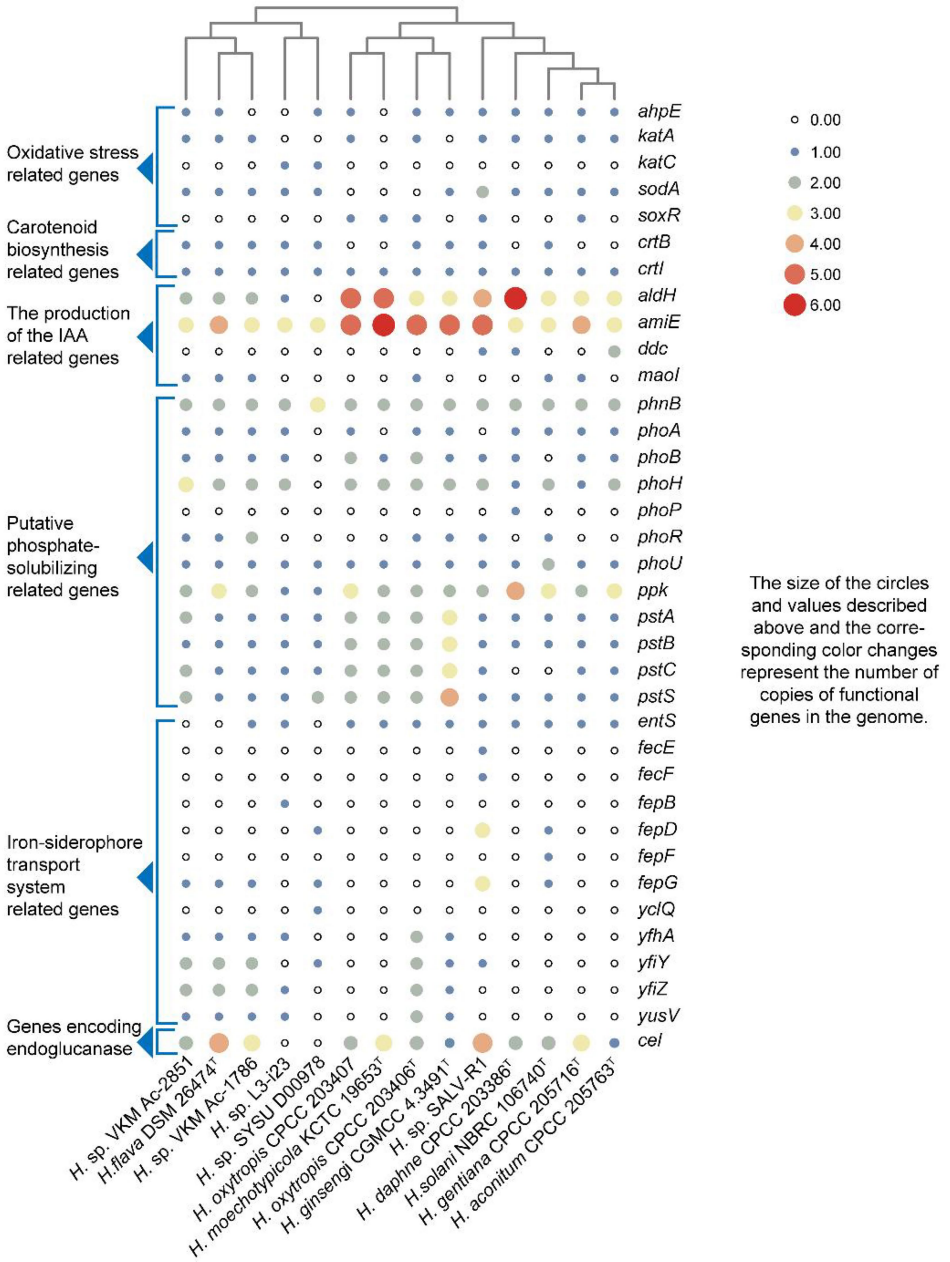
Heatmap of putative functional genes predicted from the 14 genomes of the genus *Herbiconiux* according to the copy number of the genes from the KEGG annotation.

The whole genomes of strains CPCC 205763^T^, CPCC 203386^T^, CPCC 205716^T^, CPCC 203406^T^, CPCC 203407, *H. flava* DSM 26474^T^, *H. ginsengi* CGMCC 4.3491^T^, *H. moechotypicola* KCTC 19653^T^, *H. solani* NBRC 106740^T^, *Herbiconiux* sp. L3-i23, *Herbiconiux* sp. SALV-R1, *Herbiconiux* sp. SYSU D00978, *Herbiconiux* sp. VKM Ac-1786 and *Herbiconiux* sp. VKM Ac-2,851 contained 3,952, 5,647, 3,674, 3,987, 3,945, 3,678, 4,594, 4,087, 3,688, 3,057, 4,305, 2,646, 3,742, and 4,143 genes, respectively. The detailed genomic characteristics of these 14 strains were summarized in Supplementary Table S4. In the 14 genomes of the genus *Herbiconiux*, the putative encoding genes for alkyl hydroperoxide reductase E (*ahpE*), catalase (*katA, katC*), manganese catalase (*ydbD*), redox-sensitive transcriptional activator SoxR (*soxR*) and superoxide dismutase (*sodA*) were retrieved. These enzymes might help these microorganisms to relieve the stress of excessive oxygen anion concentration that released from the medicinal plants into the environments. The carotenoid biosynthesis related genes (*crtB* and *crtI*), indole-3-acetic acid producing related genes (*aldH*, *amiE*, *ddc maoI*), iron-siderophore transport system permease protein associated encoding genes (*entS*, *fecE*, *fecF*, *fepB*, *fepD*, *fepG*, *yclQ*, *yfhA*, *yfiY*, *yfiZ*, and *yusV*) and phosphate-solubilizing encoding genes (*phnB*, *phoA*, *phoB*, *phoH*, *phoP*, *phoR*, *phoU*, *ppk, pstA*, *pstB*, *pstC*, and *pstS*) were also sorted (Figure 3).

### Pangenome analysis of the genus *Herbiconiux*

**Figure 4:**
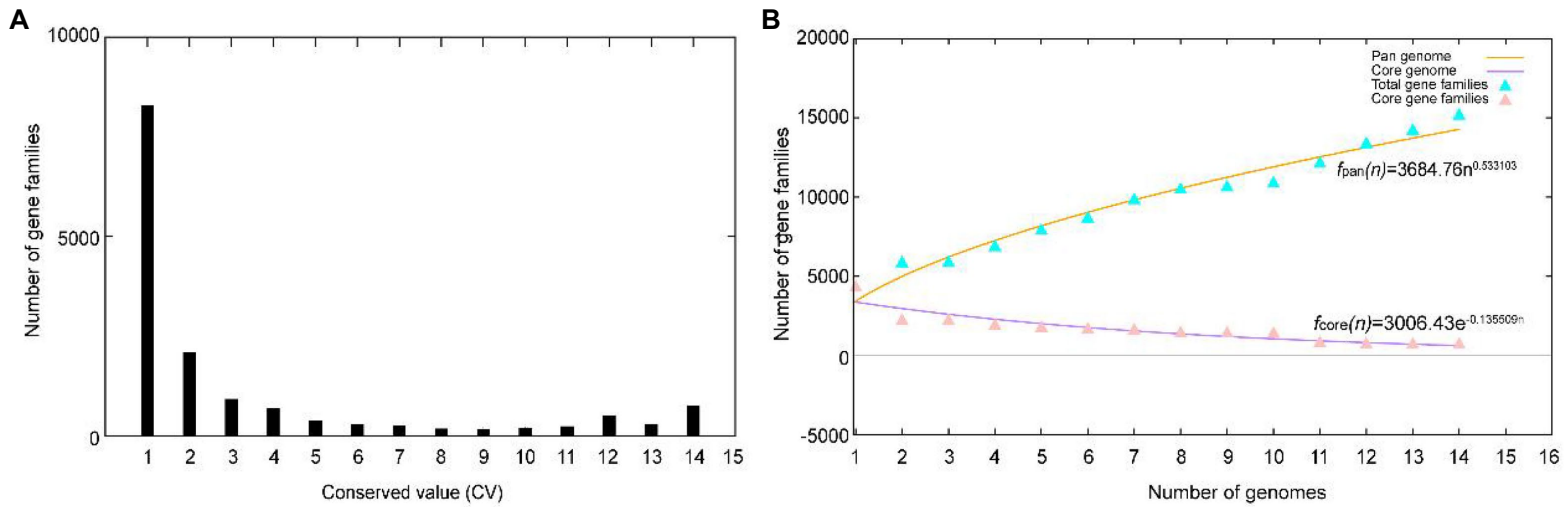
Overview of the pan-genomic results generated by BPGA using the 14 genomes of the genus *Herbiconiux*. **(A)** The gene family frequency spectrum. **(B)** The pan genome profile trends of the genus *Herbiconiux* obtained using clustering tools USEARCH.

A total of 51,271 protein-coding genes (Table 2) were sorted from the genomes of these 14 strains of the genus *Herbiconiux*, which were divided into 15,209 homologous families by cluster analysis. According to the presence or absence of a certain homologous gene in these 14 genomes, we defined the homologous gene families conservation values (HGFCV, be abbreviated to CV). Each homologous family was given a conserved value (CV) based on its frequency of occurrence in each of the 14 genomes. Histograms were constructed according to different CVs (Figure 4A). In the pan-genome profile of the genus *Herbiconiux*, there were a total of 753 core genes commonly shared by these 14 strains (CV = 14), accounting for about 5.0% of the total number of homologous gene families. The accessory genes (6,160 genes; CV = 2 ~ 13) accounted for about 40.5% of the homologous gene families in the genus *Herbiconiux*. The proportion of the unique genes (8,296 genes; CV = 1) was about 54.5%.

**Table 2:** Distribution of core, accessory, unique and exclusively absent genes among the genomes of 14 strains of the genus *Herbiconiux* using USEARCH clustering tool.

Organism	No. of core genes	No. of accessory genes	No. of unique genes	No. of exclusively absent genes
1	753	2,550	1,051	3
2	753	2,998	46	1
3	753	3,004	21	1
4	753	2,495	293	1
5	753	2,468	583	4
6	753	2,158	591	13
7	753	2,380	961	3
8	753	2,572	373	6
9	753	2,503	123	4
10	753	2,349	229	10
11	753	751	1,079	142
12	753	1,001	1,171	84
13	753	2,703	811	1
14	753	2,222	964	6

1, CPCC 203386^T^; 2, CPCC 203406^T^; 3, CPCC 203407; 4, CPCC 205716^T^; 5, CPCC 205763^T^;6, *H*. *solani* NBRC 106740^T^; 7, *Herbiconiux* sp. SALV-R1; 8, *Herbiconiux* sp. VKM Ac-2,851; 9, *H. flava* NBRC 16403^T^; 10, *Herbiconiux* sp. VKM Ac-1786; 11, *Herbiconiux* sp. SYSU D00978; 12, *Herbiconiux* sp. L3-i23; 13, *H. ginsengi* KCTC 19440^T^; 14, *H*. *moechotypicola* KCTC 19653^T^.

The relationship between the pan-genome size and the number of genomes of the genus, and the relationship between the number of core genes and the number of genomes were deduced (Figure 4B) by using all the protein sequences extracted from these 14 strains of the genus *Herbiconiux*. The functional relationship between pan-genome size (f*_pan_*) and the number of genomes (n) was obtained by fitting, as follows:

$$f_{\mathrm{pan}}\;\left(n\right)=3684.76\times n^{0.533103}$$

Meanwhile, the functional relationship between the number of core genes (*f_core_*) and the number of genomes (n) was obtained by fitting, as follows:

$$f_{\mathrm{core}}\;\left(n\right)=3006.43\times e^{-0.135509\;n}$$

It could be observed from the pan-genome fitting curve in the Figure 4B that, with the increasing number of sequenced genomes, the pan-genome size become larger and larger, instead of tending to a plateau. Accordingly, it could be inferred that the pan-genome of the genus *Herbiconiux* is the open type, which suggests that the genus *Herbiconiux* has a strong ability to accept horizontal gene transfer events.

**Figure 5:**
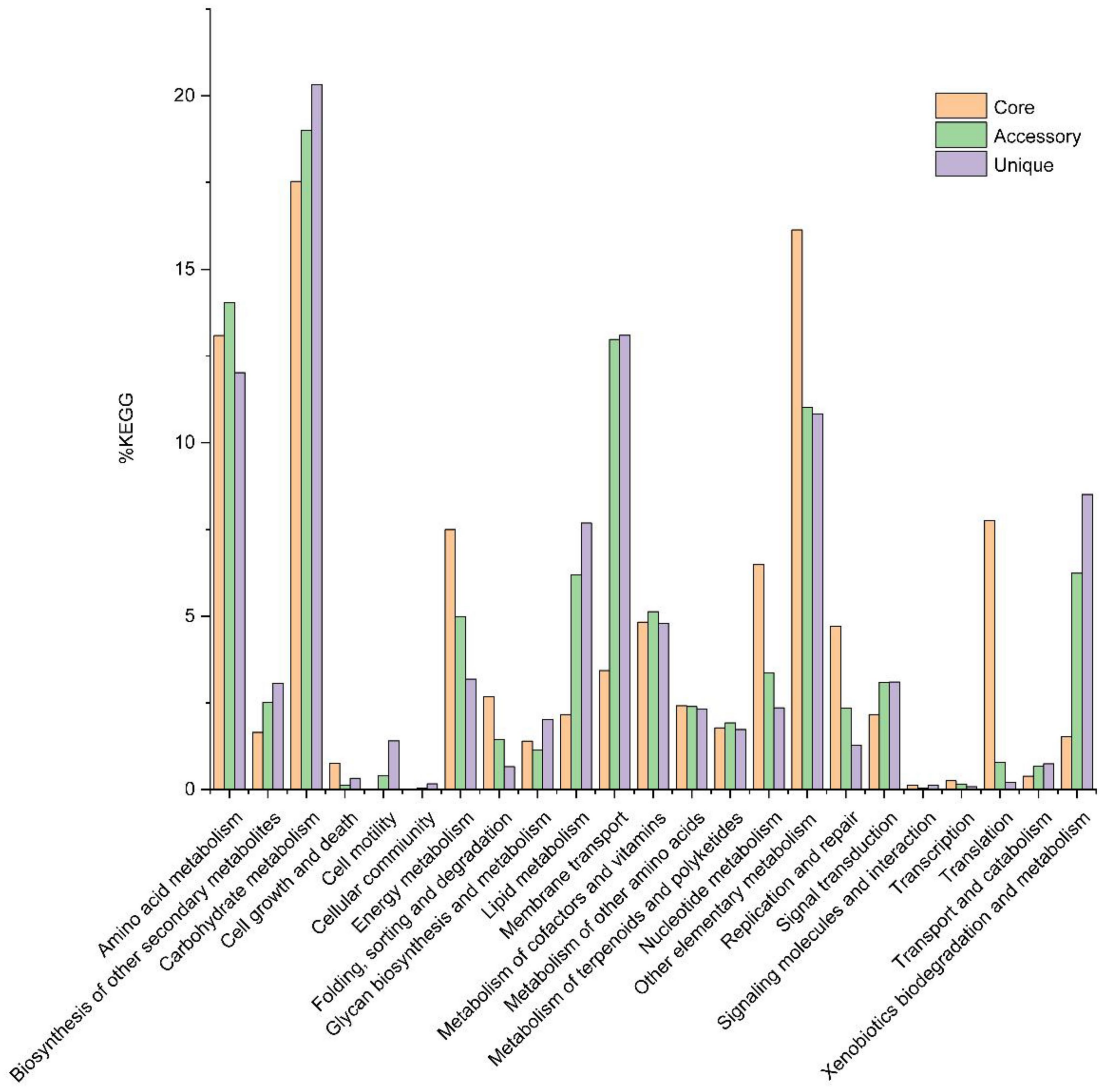
The assigned metabolic pathways associated with the core, accessory and unique genes among the genus *Herbiconiux* from the KEGG database (14 strains).

Out of 15,209 genes (clusters), BPGA could map 6,136 (40.3%) to KEGG (Kyoto Encyclopedia of Genes and Genomes) pathways, i.e., core gene (822, 13.4%), accessory genes (2,735, 44.6%) and unique genes (2,579, 42.0%). After filtering some KEGG pathway related to eukaryotes, we obtained an overview on the metabolic pathway (>1%) corresponding to the gene(s) in the pan-genome of the genus *Herbiconiux*. A large number of core genes (787) were involved in carbohydrate metabolism (17.5%), some other elementary metabolism (biosynthesis of amino acids, 8.4%; carbon metabolism, 5.0%; 2-oxocarboxylic acid metabolism, 2.0%; fatty acid metabolism, 0.6% and degradation of aromatic compounds, 0.1%; 16.1%), amino acid metabolism (13.1%), translation (7.8%), energy metabolism (7.5%), nucleotide metabolism (6.5%), metabolism of cofactors and vitamins (4.8%), replication and repair (4.7%), membrane transport (3.4%), folding, sorting and degradation (2.7%), metabolism of other amino acids (2.4%), signal transduction (2.2%), lipid metabolism (2.2%), metabolism of terpenoids and polyketides (1.8%), biosynthesis of other secondary metabolites (1.7%), xenobiotics biodegradation and metabolism (1.5%), and glycan biosynthesis and metabolism (1.4%). Accessory and unique genes appeared to be enriched in carbohydrate metabolism, amino acid metabolism, membrane transport, as well as other elementary metabolism. Among the accessory genes (2,551), the major portion of genes seemed related to carbohydrate metabolism (19.0%), amino acid metabolism (14.0%), membrane transport (13.0%), some other elementary metabolism (carbon metabolism, 4.7%; biosynthesis of amino acids, 3.1%; fatty acid metabolism, 1.5%; degradation of aromatic compounds, 1.3% and 2-oxocarboxylic acid metabolism, 0.5%; 11.0%), xenobiotics biodegradation and metabolism (6.2%), lipid metabolism (6.2%), metabolism of cofactors and vitamins (5.1%), energy metabolism (5.0%), nucleotide metabolism (3.4%), signal transduction (3.1%), biosynthesis of other secondary metabolites (2.5%), metabolism of other amino acids (2.4%), replication and repair (2.4%), metabolism of terpenoids and polyketides (1.9%), folding, sorting and degradation (1.5%) and glycan biosynthesis and metabolism (1.1%). Unique genes (2,421) seemed to be mainly enriched in carbohydrate metabolism (20.3%), membrane transport (13.1%), amino acid metabolism (12.0%), some other elementary metabolism (carbon metabolism, 3.9%; biosynthesis of amino acids, 3.2%; fatty acid metabolism, 1.7%; degradation of aromatic compounds, 1.3% and 2-oxocarboxylic acid metabolism, 0.7%; 10.8%), xenobiotics biodegradation and metabolism (8.5%), lipid metabolism (7.7%), metabolism of cofactors and vitamins (4.8%), energy metabolism (3.2%), signal transduction (3.1%), biosynthesis of other secondary metabolites (3.1%), nucleotide metabolism (2.4%), metabolism of other amino acids (2.3%), glycan biosynthesis and metabolism (2.0%), metabolism of terpenoids and polyketides (1.7%), cell motility (1.4%) and replication and repair (1.3%; Figure 5).

### Secondary metabolite biosynthesis gene clusters analysis

The results from antiSMASH database showed that in these 14 strains of the genus *Herbiconiux*, three to eight secondary metabolite gene clusters with moderate similarities to previously described secondary metabolite biosynthetic gene clusters were retrieved. These gene clusters exhibited 1–100% similarities to previously reported secondary metabolite biosynthetic gene clusters, such as carotenoid, pyoverdin, microansamycin, tiancimycin, rustmicin, herboxidiene, divergolide A/ divergolide B/ divergolide C/ divergolide D, cypemycin, alkylresorcinol gene clusters and other unidentified secondary metabolite clusters attributable to NAPAA, redox-cofactor, RiPP-like, RRE-containing, NRPS-like, bottromycin, and T3PKS types, respectively (Supplementary Table S5).

## Discussion

Obviously, the 16S rRNA gene sequences alignment and the phylogenetic analysis indicated these five isolates affiliated to the genus *Herbiconiux*, which was supported by the chemotaxonomic traits. The genome relatedness indexes, i.e., values of ANI and dDDH between strains CPCC 205763^T^, CPCC 203386^T^, CPCC 205716^T^, CPCC 203406^T^, and all the type strains of the validly named species in the genus *Herbiconiux* well differentiated them from each other. The ANI and dDDH values between strain CPCC 203406^T^ and CPCC 203407 suggested to classify these two strains into a same species. Integrated the morphological properties, physiologic characteristics, chemotaxonomic profiles and phylogenetic analysis of these five strains, it is reasonable to propose four novel species of the genus *Herbiconiux,* i.e., *Herbiconiux aconitum* sp. nov. with CPCC 205763^T^ as the type strain, *Herbiconiux daphne* sp. nov. with CPCC 203386^T^ as the type strain, *Herbiconiux gentiana* sp. nov. with CPCC 205716^T^ as the type strain, and *Herbiconiux oxytropis* sp. nov. with CPCC 203406^T^ as the type strain.

**Figure 6:**
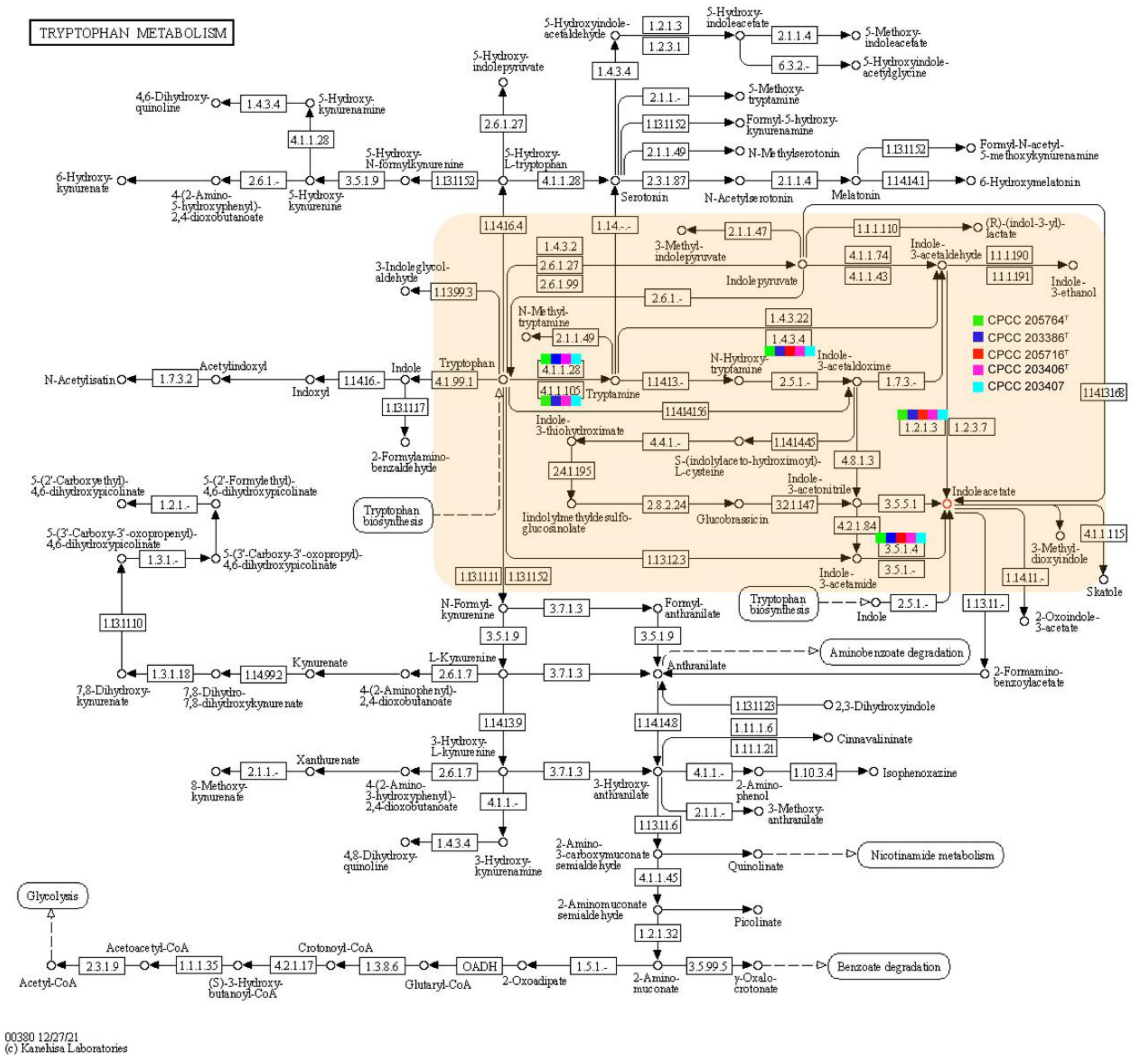
Putative overview of indole-3-acetic acid-producing pathway in the tryptophan metabolism pathways of the strains CPCC 205763^T^, CPCC 203386^T^, CPCC 205716^T^, CPCC 203406^T^, and CPCC 203407. The squares colored in green, blue, red, pink and light blue represents the strain CPCC 205763^T^, CPCC 203386^T^, CPCC 205716^T^, CPCC 203406^T^, and CPCC 203407, respectively.

Rhizospheric and endophytic microorganisms live together with the plants for a time, and co-evolve with the host plant, where microbes and plants shared the common special mico-ecosystem, so that they form a mutually beneficial symbiotic relationship during the evolution process. On the one hand, plants provide photosynthetic products and minerals for the growth of these microbes. In turn, once a kind of beneficial bacteria colonizes in a niche associated with a plant, it can influence the physiologic traits of the plant through various mechanisms. In this study, strains CPCC 205763^T^, CPCC 203386^T^, CPCC 205716^T^, CPCC 203406^T^, and CPCC 203407 were isolated from different ecosystems associated with four kinds of Chinese traditional medicinal plants, *Aconitum carmichaelii, Daphne aurantiaca, Gentiana rigescens* and *Oxytropis falcate*, respectively. As well, the strains *Herbiconiux flava* DSM 26474^T^, *Herbiconiux ginsengi* CGMCC 4.3491^T^, *Herbiconiux solani* NBRC 106740^T^, *Herbiconiux* sp. SALV-R1, *Herbiconiux* sp. VKM Ac-1786 and *Herbiconiux* sp. VKM Ac-2,851 were reported to inhabit the niches associated with plants. Based on the genomic information, we summarized genetic characteristics of these *Herbiconiux* spp. The detailed phenotypic properties illustrated the abilities of these strains to make contribution to their associated medicinal plants. For instance, indole-3-acetic acid (IAA), a kind of plant hormone, plays an important role in plant-microbial interactions, especially, in promoting the growth of plants. The phenotypic assays confirmed that strains CPCC 205763^T^, CPCC 203386^T^, CPCC 205716^T^, CPCC 203406^T^, and CPCC 203407 could produce IAA. In the genomic category of these five strains, the related encoding genes involved in the IAA-producing pathway were retrieved. For instance, the encoding genes for aldehyde dehydrogenase (EC 1.2.1.3; *aldH*), amidase (Unwin et al., 2004), aromatic-L-amino-acid/L-tryptophan decarboxylase (*ddc*) and monoamine oxidase (MAO, *aofH*). What’s more, in the core genome of the genus *Herbiconiux*, the amidase coding gene (Unwin et al., 2004) was retrieved. In addition, according to the distribution of IAA-producing genes in the five strains, the pathway of IAA production by the annotation of the KEGG database could be predicted (Figure 6).

The metabolites produced by rhizospheric microbes or endophytes, such as IAA, can stimulate the growth and development of the plants that shared the common ecosystems with them, accordingly, improve the resistance of the plants to biotic or abiotic stresses. Therefore, some metabolites from these microorganisms are of significance for plant growth and development. In order to explore the useful metabolite-producing candidates, microbiologists are increasingly employing genome sequencing of a wide variety of such microbes. Here, we identified biosynthetic gene clusters (BGCs) by antiSMASH. From the antiSMASH results of these strains, we found that 100% (14/14) of the *Herbiconiux* genomes contain the carotenoid gene clusters (Table 3), with the 50 –66% similarities. The carotenoids belong to the isoprenoids and contain eight isoprene units, namely, tetraterpenoids. For one thing, carotenoids have the ability to absorb and transfer electrons, and play an important role in scavenging superoxide anion free radicals produced during photosynthesis (Bartley and Scolnik, 1995). In addition, carotenoids, such as astaxanthin (Chang and Xiong, 2020), fucoxanthin and zeaxanthin (Firdous et al., 2015) were reported to have anti-inflammatory functions. In this research, the four kinds of Chinese traditional medicinal plants, *Aconitum carmichaelii* (Zhao et al., 2018), *Daphne aurantiaca* (Nie et al., 2021), *Gentiana rigescens* (Zhao et al., 2015) and *Oxytropis falcate* (Chen et al., 2011), were reported to have anti-inflammatory activities. Could we suppose that these microbes isolated from these ecological niches might be endowed with the anti-inflammatory traits by their corresponding medicinal plants?

**Table 3:** Comparative results of secondary metabolite biosynthesis gene clusters predicted from the 14 strains’ genomes of the genus *Herbiconiux.*

Strain	Secondary metabolite synthesis gene clusters								
1	/	Carotenoid	/	/	/	Microansamycin	Pyoverdin	/	/
2	/	Carotenoid	/	/	/	Microansamycin	/	/	/
3	/	Carotenoid	/	/	/	Microansamycin	/	Rustmicin	Tiancimycin
4	/	Carotenoid	/	/	Herboxidiene	/	/	/	/
5	/	Carotenoid	/	Divergolide A/B/C/D	Herboxidiene	/	/	/	/
6	/	Carotenoid	Cypemycin	/	/	Microansamycin	/	/	/
7	/	Carotenoid	/	/	/	Microansamycin	/	/	/
8	/	Carotenoid	/	/	/	Microansamycin	/	Rustmicin	/
9	/	Carotenoid	/	/	/	Microansamycin	/	/	/
10	Alkylresorcinol	Carotenoid	/	/	/	Microansamycin	/	/	/
11	/	Carotenoid	/	/	/	Microansamycin	/	/	/
12	/	Carotenoid	/	/	/	Microansamycin	/	Rustmicin	/
13	/	Carotenoid	/	/	/	Microansamycin	/	rustmicin	/
14	Alkylresorcinol	Carotenoid	/	/	/	Microansamycin	/	/	/

Secondary metabolite biosynthesis gene clusters with 50% or greater similarity were highlighted in bold. 1, *Herbiconiux aconitum* sp. nov. CPCC 205763^T^; 2, *Herbiconiux daphne* sp. nov. CPCC 203386^T^; 3, *Herbiconiux gentiana* sp. nov. CPCC 205716^T^; 4, *Herbiconiux oxytropis* sp. nov. CPCC 203406^T^; 5, *Herbiconiux oxytropis* sp. nov. CPCC 203407; 6, *Herbiconiux ginsengi* CGMCC 4.3491^T^; 7, *Herbiconiux solani* NBRC 106740^T^; 8, *Herbiconiux flava* DSM 26474^T^; 9, *Herbiconiux moechotypicola* KCTC 19653^T^; 10, *Herbiconiux* sp. L3-i23; 11, *Herbiconiux* sp. SALV-R1; 12, *Herbiconiux* sp. VKM Ac-1786; 13, *Herbiconiux* sp. VKM Ac-2,851; 14, *Herbiconiux* sp. SYSU D00978. /, not detected.

From the antiSMASH results of the strain CPCC 203406^T^ and CPCC 203407, the herboxidiene gene clusters were retrieved. Herboxidiene (GEX1A) is a potent phytotoxic polyketone compound. On the one hand, as a potent splicing inhibitor in plants, GEX1A could trigger abiotic stress responses and ABA (abscisic acid) signaling in plants. Splicing stress signaling generated by GEX1A treatment is differentially regulated to ensure plant adaptation to stress conditions (AlShareef et al., 2017). On the other hand, as a novel polyketide which selectively and effectively controls several annual weed species, GEX1A could improve the biological competitiveness of the medicinal plant. In the antiSMASH results of the strain *Herbiconiux* sp. L3-i23 and *Herbiconiux* sp. SYSU D00978, the BGCs of alkylresorcinol were identified. Alkylresorcinols are phenolic lipids widely distributed in plants and bacteria. Different degrees of substitution yield various compounds that have been reported to possess antioxidant (Gliwa et al., 2011), anti-inflammatory (Wei et al., 2022), antimicrobial and antitumor activities. Given a variety of alkylresorcinols compounds have antimicrobial activity, it is possible to conclude that these compounds act as defensive agents in plants. Moreover, phenolic compounds (such as 5-n-alkylresorcinols, phlorizin, resveratrol, ferulic acid, et al.) are part of the plant’s protection system against various pests and are therefore considered natural alternatives to protective agents (Patzke and Schieber, 2018). In the annotation results of the KEGG, the spermidine/putrescine transport related genes (*potA*, *potB*, *potC*, and *potD*) were retrieved in the genomes of the new species. In plants, it has been shown that increasing polyamine levels minimizes harmful effects caused by biotic and abiotic stresses (Yoda et al., 2003).

Reactive oxygen species (ROS), generated by the plant, might be neutralized by the production of enzymes such as superoxide dismutases, catalases and alkyl hydroperoxide reductases in microorganisms. In the genomes of the strains CPCC 205763^T^, CPCC 203386^T^, CPCC 205716^T^, CPCC 203406^T^, CPCC 203407, and other members of the genus *Herbiconiux* included in our study, various antioxidant encoding genes were retrieved, such as genes coding for superoxide dismutase (*sodA*), putative manganese catalase (*ydbD*), catalase coding gene (*katA*), alkyl hydroperoxide reductase E (*ahpE*) and redox-sensitive transcriptional activator SoxR (*soxR*). The carotenoids and phytohormones mentioned above also play an important role in antioxidant activities. Therefore, the strains of the genus *Herbiconiux* may play an important role in their associated plants responding to oxidative stress.

Microorganisms promote the growth of their associated plants by obtaining nutrients from the environments. Such as the acquisition of organic phosphorus and inorganic phosphorus. At the genomic level, phosphate starvation-inducible protein coding gene (*phoH*), alkaline phosphatase coding genes (*phoA*, *phoB*), phosphate transport-related genes (*phnB*, *phoP*, *phoR*, *phoU*, *pstA*, *pstB*, *pstC*, and *pstS*) and polyphosphate kinase coding gene (*ppk*) were retrieved from all these 14 genomes. We also found some genes (*entS*) related to the production and export of the siderophore enterobactin and iron-siderophore transport system permease protein related genes (*fecE*, *fecF*, *fepB*, *fepD*, *fepF*, and *fepG*.) in the genomes of the genus *Herbiconiux* (Figure 3). Accordingly, we speculate that the new species of genus *Herbiconiux* may have beneficial effects on the growth of their associated medicinal plants, in addition to production of IAA, partially through the production of phytohormones and siderophore, solubilization of phosphorus, or by production of SOD to partly mitigate the oxidative pressure.

## Conclusion

Our research investigated the taxonomic characteristics of *Herbiconiux*, and discovered the genetic basis of *Herbiconiux* producing secondary metabolites, based on pan-genome analysis and experimental validation, specifically, the PGP function of the *Herbiconiux* spp. associated with plants. Results from this study indicated diversity of novel *Herbiconiux* members are abundant in the ecosystems associated with plants, and are a group of plants-friendly microbes. Based on these results, we expect to accumulation of sufficient *Herbiconiux* cultures from diverse ecosystems to compare the components of secondary metabolite gene clusters in different *Herbiconiux* species, and to reveal new gene elements associated with secondary metabolism and explore important secondary metabolites from *Herbiconiux*.

### Description of *Herbiconiux aconitum* sp. nov.

*Herbiconiux aconitum* (a.co.ni’ti. N.L. gen. n. *aconiti*, of *Aconitum*, a plant genus name, referring to the site related to a plant of *Aconitum carmichaelii,* from which the type strain was isolated).

Cells are aerobic, Gram-staining-positive, rod-shaped. Colonies are 0.8–1.0 mm in diameter, convex with an entire margin, glistening, viscous and bright yellow on TSA medium. Growth temperature and pH range for growth are 10–37°C and pH 7.0–8.0, with optimum growth at 28–30°C and pH 7.0. Cannot tolerate >2% (w/v) NaCl. Positive for oxidase, catalase, acid phosphatase, alkaline phosphatase, cystine arylamidase, esterase (C4), esterase lipase (C8), leucine arylamidase, naphthol-AS-BI-phosphohydrolase, valine arylamidase, *α*-galactosidase, *α*-glucosidase, *β*-galactosidase, and *β*-glucosidase, negative for lipase (C14), N-acetyl-*β*-glucosamimidase, *α*-chymotrypsin, *α*-fucosidase, *α*-mannosidase, *β*-glucuronidase and gelatin hydrolyzation. Utilizes acetic acid, acetoacetic acid, D-arabitol, D-cellobiose, dextrin, D-fructose, D-galactose, D-maltose, D-mannitol, D-mannose, D-melibiose, D-raffinose, D-salicin, D-sorbitol, D-trehalose, D-turanose, gentiobiose, glycerol, glycyl-L-proline, inosine, L-fucose, L-glutamic acid, L-lactic acid, L-rhamnose, *N*-acetyl-D-galactosamine, pectin, propionic acid, stachyose, sucrose, tween 40, *α*-D-glucose, *α*-D-lactose, *α*-hydroxy-butyric acid, and *α*-keto-butyric acid as the sole carbon source; acid is produced from arbutin, D-cellobiose, D-fructose, D-galactose, D-glucose, D-lactose, D-lyxose, D-maltose, D-mannitol, D-mannose, D-melezitose, D-raffinose, D-ribose, D-saccharose, D-tagatose, D-trehalose, D-turanose, D-xylose, esculin ferric citrate, glycerol, inulin, L-arabinose, L-rhamnose, but not from amidon, amygdalin, D-arabinose, D-arabitol, D-ardonitol, D-fucose, D-melibiose, D-sorbitol, dulcitol, erythritol, gentiobiose, glycogen, inositol, L-arabitol, L-fucose, L-xylose, mehyl-*α*-D-glucopyranoside, mehyl-*α*-D-mannopyranoside, mehyl-*β*-D-xylopyranoside, N-acetylglucosamine, potassium 2-ketogluconate, potassium 5-ketogluconate, potassium, gluconate, or xylitol. In the API 20NE test system, positive for aesculin hydrolysis, *β*-galactosidase, and assimilation of adipic acid, DL-malic acid, L-arabinose and potassium gluconate, but negative for arginine dihydrolase, D-mannitol, glucose fermentation, indole production, nitrate reduction, protease, urease, and assimilation of capric acid and phenylacetic acid. Diphosphatidylglycerol and phosphatidylglycerol are detected in the polar lipids extraction. The predominant quinone is MK-11. The fatty acid profile consists of the predominant components (> 10%) *anteiso*-C_15:0_ and summed feature 8 (C_18:1_ *ɷ*7c and/or C_18:1_ *ɷ*6c). Glucose as cell-wall sugars. The diagnostic diamino acids in the cell-wall peptidoglycan are alanine, glutamic acid and glycine. The type strain is CPCC 205763^T^ (= I19A-01430^T^ = CGMCC 1.60067^T^), isolated from a rhizosphere soil sample of the plant *Aconitum carmichaelii* collected from Xinjiang Province, north-west China. The genomic G + C content of the type strain is 68.2%.

### Description of *Herbiconiux daphne* sp. nov.

*Herbiconiux daphne* (daph’nis. N.L. gen. n. *daphnis*, of *Daphne*, a plant genus name, referring to the isolation of the type strain from a plant of *Daphne aurantiaca*).

Colonies on TSA medium are light yellow, convex with an entire margin, glistening and viscous, with 0.6–0.8 mm in diameter after 5 days at 28°C (pH 7.0). Cells are aerobic, Gram-staining-positive, rod-shaped. Growth occurs at 10–37°C and pH 7.0–8.0, with optimum at 28–32°C and pH 7.0, respectively. NaCl is not required for growth, but NaCl tolerance is up to 3.0% (w/v). Positive for oxidase and catalase reaction, negative for gelatin hydrolyzation. In the API 20NE test system, positive for aesculin hydrolysis, *β*-galactosidase, and assimilation of L-arabinose, but negative for arginine dihydrolase, D-glucose, D-mannose, D-mannitol, indole production, glucose fermentation, maltose nitrate reduction, protease, urease, and assimilation of adipic acid, capric acid, DL-malic acid, phenylacetic acid, potassium gluconate and trisodium citrate. In the API 50 CHB test system, acid is produced only from arbutin, D-cellobiose, D-galactose, D-glucose, D-maltose, D-mannitol, D-mannose, D-ribose, D-saccharose, D-trehalose, D-turanose, D-xylose, esculin ferric citrate, L-arabinose, L-fucose, L-rhamnose and salicin. Positive for acid phosphatase, esterase (C4), esterase Lipase (C8), leucine arylamidase, naphthol-AS-BI-phosphohydrolase, valine arylamidase, *α*-glucosidase, *β*-galactosidase, *β*-glucosidase and *β*-glucuronidase. Can utilize acetic acid, acetoacetic acid, D-cellobiose, D-fructose, D-galactose, D-gluconic acid, D-glucuronic acid, D-maltose, D-mannitol, D-mannose, D-salicin, D-trehalose, D-turanose, gentiobiose, glycerol, glycyl-L-proline, inosine, L-fucose, L-lactic acid, L-rhamnose, N-acetyl-D-galactosamine, pectin, p-hydroxy-phenylacetic acid, propionic acid, sucrose, tween 40, *α*-D-glucose, *α*-D-lactose, *α*-hydroxy-butyric acid and *α*-keto-butyric acid as the sole carbon source, but cannot utilize 3-methyl glucose, bromo-succinic acid, citric acid, D-arabitol, D-aspartic acid, D-fructose-6-PO_4_, D-fucose, D-galacturonic acid, D-glucose-6-PO_4_, D-lactic acid methyl ester, D-malic acid, D-melibiose, D-raffinose, D-saccharic acid, D-serine, D-sorbitol, formic acid, gelatin, glucuronamide, L-alanine, L-arginine, L-aspartic acid, L-galactonic acid, lactone, L-glutamic acid, L-histidine, L-malic acid, L-pyroglutamic acid, L-serine, methyl pyruvate, mucic acid, myo-inositol, N-acetyl neuraminic acid, N-acetyl-D-glucosamine, N-acetyl-*β*-D-mannosamine, quinic acid, *α*-Keto-glutaric acid, *β*-Hydroxy-D,L-butyric acid, *β*-Methyl-D-Glucoside or *γ*-amino-butryric acid. Diphosphatidylglycerol and phosphatidylethanolamine are detected in the polar lipids extraction. The predominant quinone is MK-11. The major cellular fatty acids are *anteiso*-C_15:0_, C_17:1_ *ɷ*9c, *anteiso*-C_17:0_ and summed feature 8 (C_18:1_ *ɷ*7c and/or C_18:1_ *ɷ*6c). Glucose as cell-wall sugars. The diagnostic diamino acids in the cell-wall peptidoglycan are alanine, glutamic acid and glycine. The type strain is CPCC 203386^T^ (=I10A-01569^T^ = DSM 24546^T^ = KCTC 19839^T^), isolated from the stem of a medicinal plant *Daphne aurantiaca* collected from Yunnan Province, south-west China. The genomic G + C content of the type strain is 65.3%.

### Description of *Herbiconiux gentiana* sp. nov.

*Herbiconiux gentiana* (gen.ti.a’nae. N.L. gen. n. *gentianae*, of *Gentiana*, a plant genus name, referring to the site related to a plant of *Gentiana rigescens,* from which the type strain was isolated).

Cells are aerobic, Gram-staining-positive, rod-shaped. Colonies are yellowish, smooth, convex and circular with diameter of 0.4–0.8 mm after 5 days on TSA medium at 28°C. Growth occurs at 4–42°C and pH 7.0–8.0, optimally at 28°C and pH 7.0. Cells are able to tolerate up to 3% NaCl (w/v) on TSA medium and grow optimally without additional NaCl. Positive for oxidase and catalase reaction, negative for gelatin hydrolyzation. In API 50CH test strips, acid is produced by D-arabitol, D-cellobiose, D-fructose, D-galactose, D-glucose, D-lyxose, D-maltose, D-mannitol, D-mannose, D-ribose, D-saccharose, D-trehalose, D-turanose, D-xylose, esculin ferric citrate, glycerol, L-arabinose and L-rhamnose, but not from amidon, arbutin, D-arabinose, D-ardonitol, D-lactose, D-melezitose, D-raffinose, D-ribose, D-sorbitol, dulcitol, erythritol, glycogen, inositol, inulin, L-arabinose, L-sorbose, mehyl-*α*-D-glucopyranoside, mehyl-*α*-D-mannopyranoside, mehyl-*β*-D-xylopyranoside, N-acetylglucosamine, potassium 2-ketogluconate, salicin, or xylitol. According to the results from the API ZYM strips, alkaline phosphatase, cystine arylamidase, esterase (C4), esterase lipase (C8), leucine arylamidase, valine arylamidase, *α*-glucosidase and *β*-glucosidase are positive; negative for lipase (C14), N-acetyl-*β-*glucosamimidase, trypsin, *α*-chymotrypsin, *α*-mannosidase and *β*-glucuronidase. In the Biolog Gen III MicroPlate system, the following carbon sources are oxidized: acetic acid, acetoacetic acid, bromo-succinic acid, citric acid, D-arabitol, D-cellobiose, dextrin, D-fructose, D-galactose, D-gluconic acid, D-malic acid, D-maltose, D-mannitol, D-mannose, D-trehalose, D-turanose, gelatin, gentiobiose, glycerol, glycyl-L-proline, inosine, L-alanine, L-aspartic acid, L-fucose, L-glutamic acid, L-lactic acid, L-malic acid, L-rhamnose, pectin, propionic acid, sucrose, tween 40, *α*-D-glucose, *α*-hydroxy-butyric acid and *β*-hydroxy-D,L-butyric acid. In the API 20NE test system, positive for aesculin hydrolysis, *β*-galactosidase, and assimilation of D-glucose, potassium gluconate and adipic acid, but negative for arginine dihydrolase, D-mannose, maltose, adipic acid, glucose fermentation, indole production, nitrate reduction, protease, urease, and assimilation of capric acid, DL-malic acid and phenylacetic acid. Diphosphatidylglycerol and phosphatidylethanolamine are detected in the polar lipids extraction. Cells contain *anteiso*-C_15:0_ and *iso*-C_16:0_ as the predominant cellular fatty acids and glucose as cell-wall sugar. The diamino acid in the cell-wall peptidoglycan are lanine, glutamic acid and glycine. The predominant quinone is MK-11. The type strain is CPCC 205716^T^ (= I21A-01427^T^ = CGMCC 1.60064^T^), isolated from a rhizosphere soil sample of the plant *Gentiana rigescens* collected from Guizhou Province, south-west China. The genomic G + C content of the type strain is 70.8%.

### Description of *Herbiconiux oxytropis* sp. nov.

*Herbiconiux oxytropis* (o.xy.tro’pis. N.L. gen. n. *oxytropis*, of *Oxytropis*, a plant genus name, referring to the isolation of the type strain from a plant of *Oxytropis falcata*).

Cells are aerobic, Gram-staining-positive and rod-shaped, form yellow-coloured colonies about 0.9–1.2 mm in diameter after growing 24 h at 28°C on TSA medium. Growth occurs at 10–37°C [optimum, 28°C and at pH 7.0–8.0 (pH 7.0)]. The range of NaCl for growth is 0–3.0% (w/v); optimum growth occurs without NaCl. Positive for oxidase, catalase reaction and gelatin hydrolyzation. In API ZYM strip test, activities of acid phosphatase, cystine arylamidase, esterase (C4), esterase lipase (C8), leucine arylamidase, naphthol-AS-BI-phosphohydrolase, valine arylamidase, *α*-glucosidase, and *β*-glucosidase are positive, but N-acetyl-*β*-glucosamimidase, *α*-chymotrypsin, *α*-fucosidase, *α*-galactosidase, *α*-mannosidase, *β*-galactosidase and *β*-glucuronidase are negative. In carbon source oxidation tests, 3-methyl glucose, acetic acid, acetoacetic acid, bromo-succinic acid, citric acid, D-arabitol, D-cellobiose, dextrin, D-fructose, D-galactose, D-gluconic acid, D-malic acid, D-maltose, D-mannitol, D-mannose, D-salicin, D-trehalose, D-turanose, gentiobiose, glycerol, glycyl-L-proline, inosine, L-alanine, L-aspartic acid, L-lactic acid, L-malic acid, L-rhamnose, methyl pyruvate, pectin, propionic acid, sucrose, tween 40, *α*-D-glucose, *α*-D-lactose, *α*-hydroxy-butyric acid, *α*-keto-butyric acid and *α*-keto-glutaric acid are oxidized. In API 50CH tests, acid is produced from D-arabitol, D-cellobiose, D-fructose, D-galactose, D-glucose, D-lyxose, D-maltose, D-mannitol, D-mannose, D-ribose, D-saccharose, D-trehalose, D-turanose, D-xylose, esculin ferric citrate, glycerol, L-arabinose and L-rhamnose, but not from amidon, amygdalin, amygdalin, arbutin, D-arabinose, D-ardonitol, D-fucose, D-lactose, D-melezitose, D-melibiose, D-raffinose, D-sorbitol, D-sorbitol, D-tagatose, dulcitol, erythritol, gentiobiose, glycogen, inositol, inulin, L-arabitol, L-fucose, L-sorbose, mehyl-*α*-D-glucopyranoside, mehyl-*α*-D-glucopyranoside, mehyl-*α*-D-mannopyranoside, mehyl-*α*-D-mannopyranoside, mehyl-*β*-D-xylopyranoside, N-acetylglucosamine, N-acetylglucosamine, potassium 2-ketogluconate, potassium 5-ketogluconate, potassium gluconate, salicin, or xylitol. In the API 20NE test system, positive for aesculin hydrolysis, *β*-galactosidase, and assimilation of D-glucose, D-mannitol, maltose, potassium gluconate and trisodium citrate, but negative for arginine dihydrolase, glucose fermentation, indole production, nitrate reduction, protease, urease, and assimilation of adipic acid, capric acid, D-mannose, DL-malic acid, L-arabinose and phenylacetic acid. Diphosphatidylglycerol and phosphatidylglycerol are detected in the polar lipids extraction. The predominant quinone is MK-11. Predominant cellular fatty acids are *anteiso*-C_15:0_, *anteiso*-C_17:0_ and *iso*-C_16:0_. Glucose, rhamnose and ribose as cell-wall sugars. The diagnostic diamino acids in the cell-wall peptidoglycan are alanine, glutamic acid and glycine. The type strain is CPCC CPCC 203406^T^ (=I10A-02268^T^ = DSM 24549^T^ = KCTC 19840^T^), isolated from the leaf of a medicinal plant *Oxytropis falcata* collected from Tibet, west China. The genomic G + C content of the type strain is 70.1%.

## Author’s note

The 16S rRNA gene sequences and the whole genome shotgun projects of the strains have been deposited at DDBJ/ENA/GenBank under the accession numbers as follows: CPCC 205763^T^ (OP279615; JANLCM000000000), CPCC 203386^T^ (JX273670; JANLCJ000000000), CPCC 205716^T^ (OP279534; JANTEZ000000000), CPCC 203406^T^ (JX273672; JANLCL000000000), and CPCC 203407 (JX273671; JANLCK000000000).

## Data availability statement

The datasets presented in this study can be found in online repositories. The names of the repository/repositories and accession number(s) can be found in the article/Supplementary material.

## Author contributions

YD, Z-MJ, X-FH, and JS carried out the experiments. YD, Z-MJ, X-FH, and Y-QZ conceived the research, analyzed the data, and prepared the manuscript. L-YY and W-HL collected the samples. All authors contributed to the article and approved the submitted version.

## Funding

This research was supported by CAMS Innovation Fund for Medical Sciences (CIFMS, 2021-I2M-1-055), National Natural Science Foundation of China (32170021 and 81960712), Beijing Natural Science Foundation (5212018), Key project at central government level-the ability establishment of sustainable use for valuable Chinese medicine resources (2060302), and the National Infrastructure of Microbial Resources (NIMR-2021-3).

## Conflict of interest

The authors declare that the research was conducted in the absence of any commercial or financial relationships that could be construed as a potential conflict of interest.

## Publisher’s note

All claims expressed in this article are solely those of the authors and do not necessarily represent those of their affiliated organizations, or those of the publisher, the editors and the reviewers. Any product that may be evaluated in this article, or claim that may be made by its manufacturer, is not guaranteed or endorsed by the publisher.

## Footnotes

^1^ https://lpsn.dsmz.de/genus/herbiconiux

^2^ http://eztaxon-e.ezbiocloud.net

^3^ http://www.cbcb.umd.edu/software/glimmer/

^4^ https://www.ebi.ac.uk/interpro/

^5^ https://www.uniprot.org/

## Abbreviations

ANI: average nucleotide identity
dDDH: digital DNA–DNA hybridization
IAA: indole-3-acetic acid
DPPH: 2,2-diphenyl-1-picrylhydrazyl
