# Corrigendum: Pangenome analysis of the genus *Herbiconiux* and proposal of four new species associated with Chinese medicinal plants

**DOI:** 10.3389/fmicb.2023.1295710

**Published:** 2024-01-05

**Authors:** Yang Deng, Zhu-Ming Jiang, Xue-Fei Han, Jing Su, Li-Yan Yu, Wei-Hong Liu, Yu-Qin Zhang

**Affiliations:** ^1^Institute of Medicinal Biotechnology, Chinese Academy of Medical Sciences and Peking Union Medical College, Beijing, China; ^2^State Key Laboratory of Dao-di Herb, Beijing, China; ^3^Yunnan Provincial Key Laboratory of Entomological Biopharmaceutical R&D, Dali University, Dali, China

**Keywords:** *Herbiconiux*, pangenome, polyphasic taxonomy, medicinal plants, plant growth-promotion

In the published article, there was an error in the Acknowledgments statement. “Prof. Aharon Oren (The Institute of Life Sciences, The Hebrew University of Jerusalem, The Edmond J. Safra Campus, Jerusalem, Israel) and prof. Bernhard Schink (Department of Biology, University of Konstanz, Konstanz, Germany)” did not propose the names for these microorganisms. Thus, the statement printed was inappropriate. The corrected Acknowledgments statement should appear below.

The authors apologize for this error and state that this does not change the scientific conclusions of the article in any way. The original article has been updated.

