# Supplementary material for: Pangenome analysis of the genus Herbiconiux and proposal of four new species associated with Chinese medicinal plants
Source: Front Microbiol. 2023 Feb 28;14:1119226. doi: 10.3389/fmicb.2023.1119226 (PMC10011130; doi:10.3389/fmicb.2023.1119226)
Supplement: Supplementary file 1 [file Data_Sheet_1.PDF]

Pangenome analysis of the genus *Herbiconiux* and proposal of four new species associated with Chinese medicinal plants

Yang Deng[1,2], Zhu-Ming Jiang[1,2‡], Xue-Fei Han[1,2‡], Jing Su[1], Li-Yan Yu[1], Wei-Hong Liu[3], Yu-Qin Zhang[1,2*]

[1]Institute of Medicinal Biotechnology, Chinese Academy of Medical Sciences & Peking Union Medical College, Beijing 100050, P. R. China

[2]State Key Laboratory of Dao-di Herb, Beijing, 100700, P. R. China

[3]Yunnan Provincial Key Laboratory of Entomological Biopharmaceutical R&D, Dali University, Dali 671003, P. R. China.

[*]Author for correspondence:

Yu-Qin Zhang

Tel: +86-10-83167110

Fax: +86-10-83167110

E-Mail: yzhang@imb.pumc.edu.cn

[‡]Zhu-Ming Jiang and Xue-Fei Han share the first author with Yang Deng.

Abbreviations: ANI, average nucleotide identity; dDDH, digital DNA-DNA hybridization; IAA, indole-3-acetic acid; DPPH, 2,2-diphenyl-1-picrylhydrazyl

FIGURE S1 | IAA standard curve and the absorbance values of the strains CPCC 205763^T, CPCC 203386^T, CPCC 205716^T, CPCC 203406^T and CPCC 203407 in their respective fermentation broth at 540 nm.

The circle colored in orange, gray, yellow, blue and green represents absorbance value of the strains CPCC 205763^T, CPCC 203386^T, CPCC 205716^T, CPCC 203406^T and CPCC 203407, respectively.

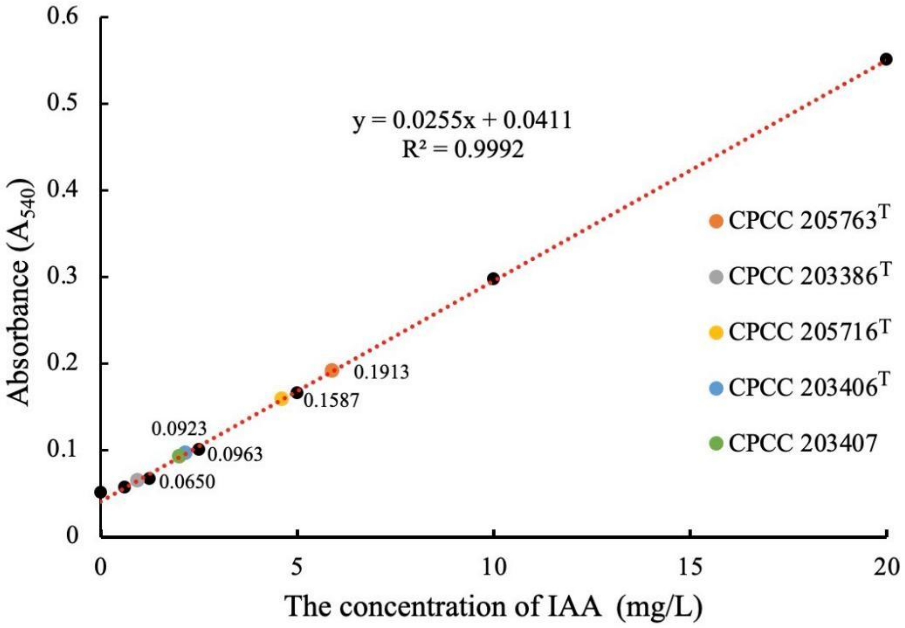

FIGURE S2 | The radical scavenging activity standard curve based on vitamin C and the radical scavenging activities of strains CPCC 205763^T, CPCC 203386^T, CPCC 205716^T, CPCC 203406^T and CPCC 203407 measured by DPPH method. The circle colored in gray, yellow, blue, green and dark blue represents the strain CPCC 205763^T, CPCC 203386^T, CPCC 205716^T, CPCC 203406^T and CPCC 203407, respectively.

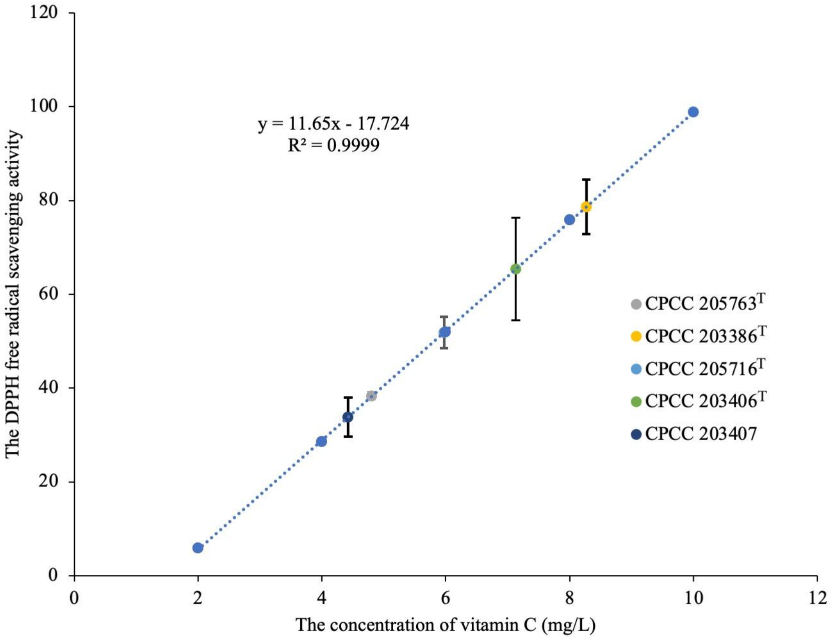

FIGURE S3 | Polar lipid profiles for strains CPCC 205763^{T}, CPCC 203386^{T}, CPCC 205716^{T}, CPCC 203406^{T} and CPCC 203407 after separation by two-dimensional TLC.

(a) was detected by spraying with molybdatophosphoric acid reagent; (b) was detected by spraying with molybdenum blue stain reagent; (c) was detected by spraying with p-anisaldehyde stain reagent.

DPG, diphosphatidylglycerol; PG, phosphatidylglycerol; GL, glycolipid.

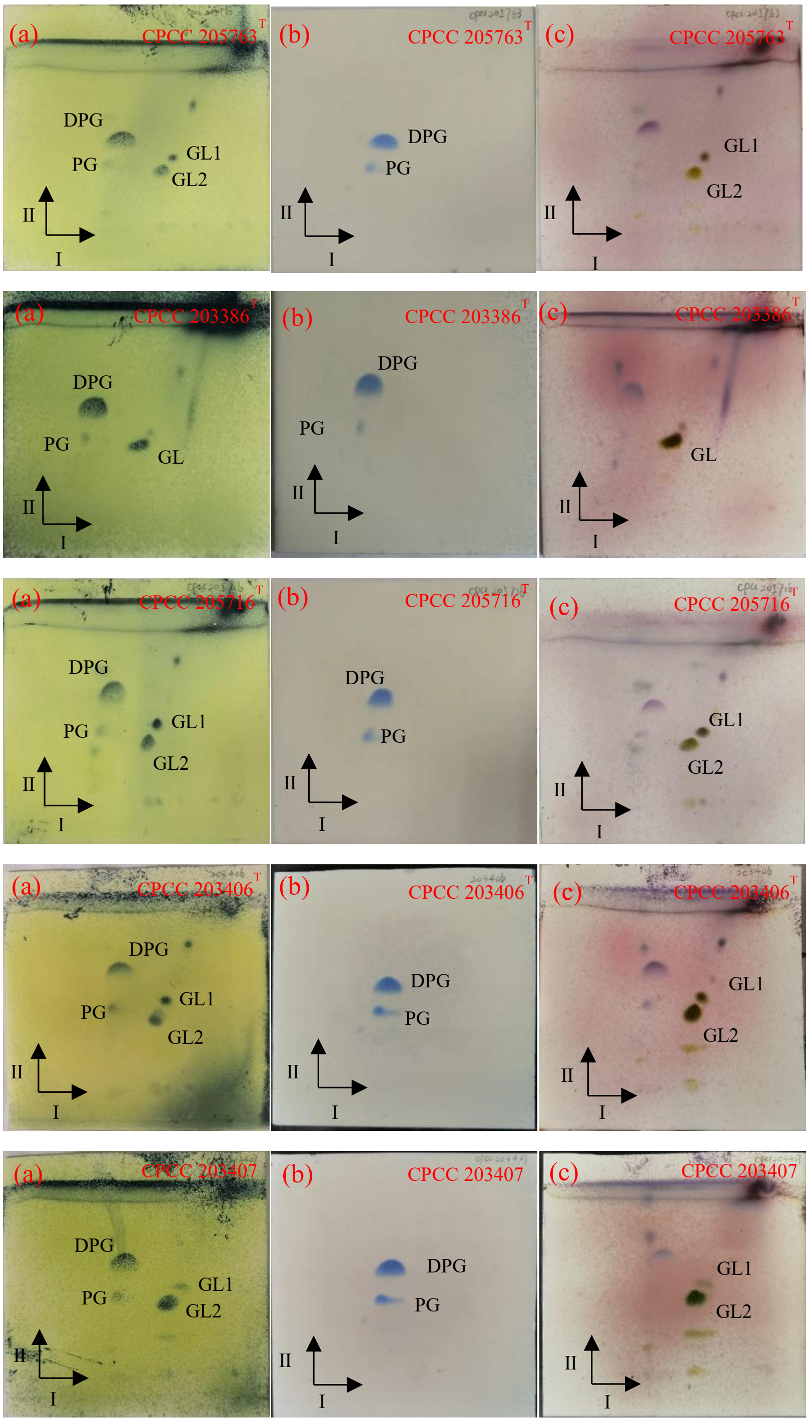

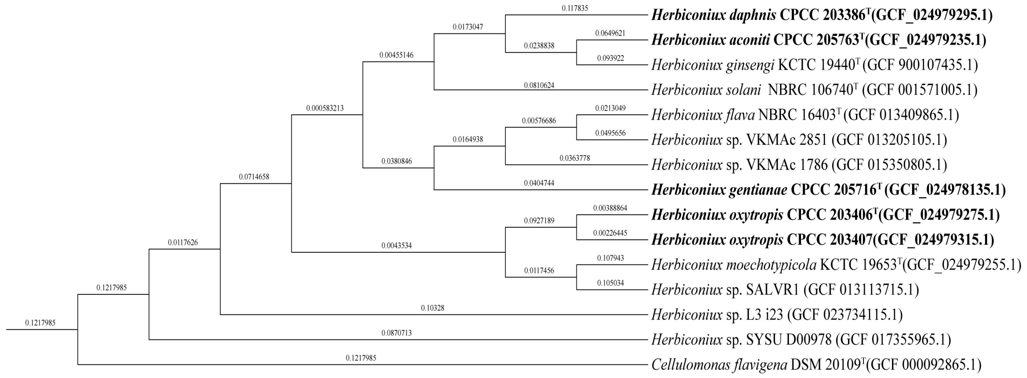

FIGURE S4 | Phylogenetic tree constructed by BPGA showing the relationship of the newly proposed species with other species of the genus *Herbiconiux* based on binary gene presence/absence matrix (pan-matrix).

Table S1 | Cellular fatty acids contents of strains CPCC 205763^{T}, CPCC 203386^{T}, CPCC 205716^{T}, CPCC 203406^{T}, CPCC 203407 and the four type strains of the genus *Herbiconiux.*

Strains: 1, CPCC 205763^{T}; 2, *H. ginsengi* KCTC 19440^{T}; 3, CPCC 203386^{T}; 4, *H. flava* NBRC 16403^{T}; 5, CPCC 205716^{T}; 6, CPCC 203406^{T}; 7, CPCC 203407; 8, *H. solani* NBRC 106740^{T}; 9, *H. moechotypicola* KCTC 19653^{T}.

Data were from this study except (a) Undine et al. 2011; (b) Kim et al. 2012. -, not detected.

Fatty acid (%)	1	2	3	4	5	6	7	8[a]	9[b]
Antesio-branched fatty acid									
anteiso- $C_{15:0}$	41.1	33.3	26.2	52.5	63.7	70.3	62.0	45.1	34.6
anteiso- $C_{17:0}$	9.1	10.6	11.1	15.6	9.9	12.6	12.1	14.3	29.9
iso-branched fatty acid									
iso- $C_{14:0}$	1.0	0.7	-	1.1	3.0	2.2	2.8	1.0	-
iso- $C_{15:0}$	2.3	0.7	-	12.5	0.7	1.0	-	15.9	1.5
iso- $C_{16:0}$	5.3	7.7	8.5	10.1	20.6	10.7	12.3	8.9	17.1
iso- $C_{17:0}$	-	-	-	2.1	-	-	-	3.4	-
iso- $C_{20:0}$	-	-	-	-	-	-	1.1	-	-
Straight-chain fatty acid									
$C_{14:0}$	-	-	-	0.7	-	-	-	-	-
$C_{16:0}$	1.1	1.3	7.6	3.0	0.8	0.7	-	-	2.2
$C_{18:0}$	0.6	-	-	-	-	-	-	-	-
Hydroxy fatty acids									
$C_{10:0}$ 3OH	-	-	-	-	-	-	0.9	-	-
$C_{12:0}$ 2OH	-	-	-	-	-	-	1.5	-	-
$C_{14:0}$ 2OH	0.6	-	-	-	-	-	-	-	-
iso- $C_{14:0}$ 3OH	2.6	-	-	-	-	-	-	-	-
$C_{16:0}$ N alcohol	-	-	-	0.6	-	-	-	-	-
Unsaturated									
anteiso- $C_{15:1}$ A	0.7	-	1.3	-	-	-	-	-	-
$C_{16:1}$ $\varpi 11c$	1.7	1.2	-	-	-	-	-	-	-
$C_{17:1}$ ϖ9c	-	-	25.0	-	-	-	-	-	-
$C_{18:1}$ $\varpi 9c$	-	-	5.8	1.0	-	-	-	-	-
Others									
Sum Feature 2 ($C_{12:0}$ aldehyde)	0.5	-	-	-	-	-	-	-	-
Sum Feature 3 ($C_{16:1}\varpi 7c$/$C_{16:1}\varpi 6c$)	0.7		-	-	-	-	1.2	-	-
Sum Feature 4 (anteiso-$C_{17:1}$B/iso-$C_{17:1}$ I)	3.2	0.5	1.9	-	0.7	0.8	4.6	-	-
Sum Feature 8 ($C_{18:1}\varpi 7c$/$C_{18:1}\varpi 6c$)	28.1	43.6	12.7	-	-	1.5	1.7	-	11.5

Table S2 | The 16S rRNA gene sequence similarities (%) between strains CPCC 205763^T, CPCC 203386^T, CPCC 205716^T, CPCC 203406^T, CPCC 203407 and other closely related strains of the genus *Herbiconiux*.

Strains: 1, CPCC 205763^T; 2, CPCC 203386^T; 3, CPCC 205716^T; 4, CPCC 203406^T; 5, CPCC 203407; 6, *H. ginsengi* KCTC 19440^T; 7, *H. flava* NBRC 16403^T; 8, *Herbiconiux* sp. VKM Ac-2851; 9, *Herbiconiux* sp. VKM Ac-1786; 10, *H. solani* NBRC 106740^T; 11, *H. moechotypicola* KCTC 19653^T; 12, *Herbiconiux* sp. SALV-R1; 13, *Herbiconiux* sp. SYSU D00978; 14, *Herbiconiux* sp. L3-i23. The 16S rRNA gene sequence similarities (%) higher than 98.65 % were colored in red.

	1	2	3	4	5	6	7	8	9	10	11	12	13	14
1	100.0													
2	97.6	100.0												
3	98.0	98.0	100.0											
4	98.6	97.6	98.8	100.0										
5	98.6	97.6	98.8	100.0	100.0									
6	99.3	97.7	98.0	98.6	98.6	100.0								
7	97.8	97.9	99.7	98.8	98.8	97.8	100.0							
8	98.4	98.0	100.0	99.6	99.6	98.4	99.8	100.0						
9	98.0	98.0	100.0	98.8	98.8	97.9	99.7	100.0	100.0					
10	98.4	97.4	98.4	98.8	98.8	98.2	98.3	98.7	98.4	100.0				
11	97.9	97.4	97.5	97.8	97.8	97.8	97.7	98.3	97.4	97.4	100.0			
12	98.1	97.2	98.9	98.9	98.9	97.9	98.9	99.3	98.8	98.0	98.3	100.0		
13	96.2	95.7	95.8	96.2	96.2	96.2	95.9	96.3	95.7	96.3	96.8	95.8	100.0	
14	97.0	95.7	95.9	96.7	96.7	96.6	96.0	96.1	99.0	96.3	97.3	96.2	95.6	100.0

TABLE S3 | Average nucleotide identity (ANI) and digital DNA–DNA hybridization (dDDH) (%) values between strains CPCC 205763^T, CPCC 203386^T, CPCC 205716^T, CPCC 203406^T, CPCC 203407 and other closely related strains of the genus *Herbiconiux*.

1, CPCC 205763^T; 2, CPCC 203386^T; 3, CPCC 205716^T; 4, CPCC 203406^T; 5, CPCC 203407; 6, *H. ginsengi* KCTC 19440^T; 7, *H. flava* NBRC 16403^T; 8, *Herbiconiux* sp. VKM Ac-2851; 9, *Herbiconiux* sp. VKM Ac-1786; 10, *H. solani* NBRC 106740^T; 11, *H. moechotypicola* KCTC 19653^T; 12, *Herbiconiux* sp. SALV-R1; 13, *Herbiconiux* sp. SYSU D00978; 14, *Herbiconiux* sp. L3-i23. The values of ANI higher than 95 % and and dDDH higher than 70 % were colored in red, respectively.

Strain	ANI value (%)					dDDH value (%)				
	1	2	3	4	5	1	2	3	4	5
1	100.0	81.2	79.3	79.4	79.2	100.0	26.5	24.7	23.4	23.5
2	81.2	100.0	80.3	80.3	80.1	26.5	100.0	23.8	23.4	23.4
3	79.3	80.3	100.0	79.8	79.7	24.7	23.8	100.0	28.0	28.1
4	79.4	80.3	79.8	100.0	100.0	23.4	23.4	28.0	100.0	99.9
5	79.2	80.1	79.7	100.0	100.0	23.5	23.4	28.1	99.9	100.0
6	86.4	81.6	79.9	79.6	79.5	42.2	24.8	24.9	24.3	24.4
7	79.4	80.3	88.7	80.0	79.8	25.6	24.8	68.1	28.8	28.9
8	79.4	80.2	88.8	79.6	79.8	24.5	23.9	59.8	26.8	26.9
9	79.6	80.4	89.2	79.9	80.0	25.5	24.7	37.5	27.9	28.0
10	79.7	80.3	80.3	79.6	79.6	25.3	24.3	29.2	26.6	26.7
11	78.9	79.5	79.4	79.4	79.7	22.4	22.2	26.0	26.3	26.4
12	79.3	80.0	80.0	80.0	80.1	23.9	22.7	27.5	26.5	26.6
13	74.3	74.6	75.1	74.7	74.7	15.2	15.3	16.9	16.4	16.5
14	74.1	74.3	74.3	73.8	74.0	15.0	14.8	15.7	15.6	15.6

Table S4 | The information of the genomes included in the pan-genome study.

The assembled genomic data of CPCC 205763^T, CPCC 203386^T, CPCC 205716^T, CPCC 203406^T, CPCC 203407 and *H. moechotypicola* KCTC 19653^T were from this study; the assembled genomic data of *H. flava* NBRC 16403^T, *H. ginsengi* KCTC 19440^T, *H. solani* NBRC 106740^T, *Herbiconiux* sp. L3-i23, *Herbiconiux* sp. SALV-R1, *Herbiconiux* sp. SYSU D00978, *Herbiconiux* sp. VKM Ac-1786 and *Herbiconiux* sp. VKM Ac-2851 were from NCBI RefSeq database.

Strain	GenBank accession number	RefSeq assembly accession number	Genome size (Mb)	G+C (%)	N50 (bp)	Qualified contigs	Total gene number	Coding gene number	tRNA gene number	ncRNA number	Pseudogenes number	Sources	Completeness (%)	Contamination (%)	Strain heterogeneity (%)
CPCC 205763^T	JANLCM000000000	GCF_024979235.1	4.2	68.2	2,400,207	5	3,952	3,854	46	3	43	Rhizosphere soil, China	99.5	1.3	0
CPCC 203386^T	JANLCJ000000000	GCF_024979295.1	5.3	65.3	539,557	890	5,647	5,504	73	3	65	Plant, China	99.2	2.7	0
CPCC 205716^T	JANTEZ000000000	GCF_024978135.1	3.9	70.8	671,683	17	3,674	3,585	45	3	38	Rhizosphere soil, China	99.0	0.5	0
CPCC 203406^T	JANLCL010000000	GCF_024979275.1	4.2	70.1	114,203	74	3,987	3,903	47	3	31	Plant, China	99.0	1.6	0
CPCC 203407	JANLCK010000000	GCF_024979315.1	4.1	70.2	441,173	32	3,945	3,860	47	3	31	Plant, China	99.0	1.6	0
H. flava NBRC 16403^T	JACCBM000000000	GCF_013409865.1	3.9	71.1	3,840,394	2	3,678	3,678	45	3	164	Plant, China	99.2	0.5	0
H. ginsengi KCTC 19440^T	FNPZ00000000	GCF_900107435.1	4.9	68.4	778,937	14	4,594	4,594	47	3	56	Plant, China	99.5	1.9	0
H. moechotypicola KCTC 19653^T	JANLCN000000000	GCF_024979255.1	4.3	70.2	349,497	54	4,087	3,992	48	3	39	Plant, Germany	99.2	1.8	0
H. solani NBRC 106740^T	BCST00000000	GCF_001571005.1	3.9	70.2	415,719	18	3,688	3,688	49	3	40	Gut of beetles	99.0	1.4	0
Herbiconiux sp. L3-i23	AP025737	GCF_023734115.1	3.2	69.5	3,139,861	2	3,057	2,999	46	3	22	Ruins, Japan	98.5	0.5	0
Herbiconiux sp. SALV-R1	CP053344	GCF_013113715.1	4.5	70.2	4,397,911	2	4,305	4,191	46	3	40	Plant, South Korea	99.0	3.1	0
Herbiconiux sp. SYSU D00978	JAFIQW010000000	GCF_017355965.1	3.0	70.8	542,468	20	2,646	2,902	45	3	200	Sandy soil, China	99.0	0.5	0
Herbiconiux sp. VKM Ac-1786	JADKSG000000000	GCF_015350805.1	3.9	71.1	1,474,412	5	3,742	3,762	45	3	283	Plant, Russia	99.2	1.0	0
Herbiconiux sp. VKM Ac-2851	JABMLJ010000000	GCF_013205105.1	4.3	70.7	1,342,178	10	4,143	4,146	45	3	277	Plant, USA	99.2	1.0	0

Table S5 | Secondary metabolite biosynthesis gene clusters predicted from the 14 strains' genomes of the genus *Herbiconiux*.

Genomic location	Type	Secondary metabolite synthesis gene cluster	Smilarit y
**CPCC 205763^T**			
Scaffold1_1Region 1.1	terpene	carotenoid	50%
Scaffold1_1Region 1.2	NAPAA	/	/
Scaffold2_1Region 2.1	T3PKS	pyoverdin	1%
Scaffold2_1Region 2.2	betalactone	microansamycin	7%
Scaffold2_1Region 2.3	redox-cofactor	/	/
**CPCC 203386^T**			
Scaffold2Region 2.1	betalactone	microansamycin	7%
Scaffold2Region 2.2	redox-cofactor	/	/
Scaffold3Region 3.1	T3PKS	/	/
Scaffold3Region 3.2	NAPAA	/	/
Scaffold6Region 6.1	terpene	carotenoid	50%
**CPCC 205716^T**			
Scaffold1Region 1.1	betalactone	microansamycin	7%
Scaffold2Region 2.1	RRE-containing	tiancimycin	5%
Scaffold4Region 4.1	T3PKS	/	/
Scaffold6Region 6.1	terpene	carotenoid	50%
Scaffold10Region 10.1	NAPAA	rustmicin	6%
**CPCC 203406^T**			
Scaffold1Region 1.1	T3PKS	/	/
Scaffold10Region 10.1	NAPAA	herboxidiene	4%
Scaffold20Region20.1	terpene	carotenoid	50%
**CPCC 203407**			
Scaffold2Region 2.1	terpene	carotenoid	50%
Scaffold3Region 3.1	NAPAA	herboxidiene	4%
Scaffold4Region 4.1	T3PKS	/	/
Scaffold7Region 7.1	betalactone	divergolide A/ B/ C/ D	6%
***Herbiconiux ginseng* CGMCC 4.3491^T**			
NZ_FNPZ01000001Region 2.1	NAPAA	/	/
NZ_FNPZ01000001Region 2.2	terpene	carotenoid	50%
NZ_FNPZ01000002Region 3.1	T3PKS	/	/
NZ_FNPZ01000002Region 3.2	betalactone	microansamycin	7%
NZ_FNPZ01000002Region 3.3	redox-cofactor	/	/
NZ_FNPZ01000007Region 5.1	RiPP-like	/	/
NZ_FNPZ01000009Region 8.1	linaridin	cypemycin	33%
NZ_FNPZ01000012Region 9.1	RRE-containing	/	/
***Herbiconiux solani* NBRC 106740^T**			
NZ_BCST01000001Region 1.1	T3PKS	/	/
NZ_BCST01000001Region 1.2	betalactone	microansamycin	7%
NZ_BCST01000003Region 3.1	terpene	carotenoid	50%
NZ_BCST01000005Region 5.1	NRPS-like	/	/
NZ_BCST01000013Region 13.1	redox-cofactor	/	/

***Herbiconiux flava* DSM 26474^T**			
NZ_JACCBM010000001.1Region 1.1	RRE-containing	/	/
NZ_JACCBM010000001.1Region 1.2	terpene	carotenoid	50%
NZ_JACCBM010000001.1Region 1.3	betalactone	microansamycin	7%
NZ_JACCBM010000001.1Region 1.4	T3PKS	/	/
NZ_JACCBM010000001.1Region 1.5	NAPAA	rustmicin	6%
***Herbiconiux moechotypicola* KCTC 19653^T**			
Scaffold1Region 1.1	T3PKS	/	/
Scaffold2Region 2.1	terpene	carotenoid	66%
Scaffold3Region 3.1	RiPP-like	/	/
Scaffold4Region 4.1	NAPAA	/	/
Scaffold4Region 4.2	bottromycin	/	/
Scaffold6Region 6.1	betalactone	microansamycin	7%
Scaffold12Region 12.1	RRE-containing	/	/
***Herbiconiux* sp. L3-i23**			
NZ_AP025737.1Region 1.1	betalactone	microansamycin	7%
NZ_AP025737.1Region 1.2	T3PKS	alkylresorcinol	66%
NZ_AP025737.1Region 1.3	NAPAA	/	/
NZ_AP025737.1Region 1.4	terpene	carotenoid	50%
***Herbiconiux* sp. SALV-R1**			
NZ_CP053344.1Region 1.1	NAPAA	/	/
NZ_CP053344.1Region 1.2	RiPP-like	/	/
NZ_CP053344.1Region 1.3	redox-cofactor	/	/
NZ_CP053344.1Region 1.4	betalactone	microansamycin	7%
NZ_CP053344.1Region 1.5	T3PKS	/	/
NZ_CP053344.1Region 1.6	terpene	carotenoid	21%
***Herbiconiux* sp. VKM Ac-1786**			
NZ_JADKSG010000001.1Region 1.1	terpene	carotenoid	37%
NZ_JADKSG010000001.1Region 1.2	RRE-containing	/	/
NZ_JADKSG010000002.1Region 2.1	betalactone	microansamycin	7%
NZ_JADKSG010000002.1Region 2.2	T3PKS	/	/
NZ_JADKSG010000002.1Region 2.3	NAPAA	rustmicin	6%
***Herbiconiux* sp. VKM Ac-2851**			
NZ_JABMLJ010000001.1Region 1.1	betalactone	microansamycin	7%
NZ_JABMLJ010000001.1Region 1.2	T3PKS	/	/
NZ_JABMLJ010000002.1Region 2.1	terpene	carotenoid	37%
NZ_JABMLJ010000002.1Region 2.2	RRE-containing	/	/
NZ_JABMLJ010000004.1Region 4.1	NAPAA	rustmicin	6%
***Herbiconiux* sp. SYSU D00978**			
NZ_JAFIQW010000003.1Region 4.1	terpene	carotenoid	66%
NZ_JAFIQW010000003.1Region 4.2	T3PKS	alkylresorcinol	100%
NZ_JAFIQW010000007.1Region 8.1	betalactone	microansamycin	7%
